# Supplementary material for: Bidirectional terahertz frequency conversion via structural resonances at a plasma time boundary
Source: Sci Adv. 2026 Jul 31;12(31):eaed2916. doi: 10.1126/sciadv.aed2916 (PMC13426419; doi:10.1126/sciadv.aed2916)
Supplement: Supplementary file 1 — Sections S1 to S7 Figs. S1 to S4 References [file sciadv.aed2916_sm.pdf]

Science Advances
AAAS

Supplementary Materials for

Bidirectional terahertz frequency conversion via structural resonances at a plasma time boundary

Yindong Huang *et al.*

Corresponding author: Yindong Huang, yindonghuang@nudt.edu.cn; Ce Shang, shangce@aircas.ac.cn;
Xuchen Wang, xuchen.wang@hrbeu.edu.cn; Chao Chang, gwyzlzssb@pku.edu.cn;
Viktar Asadchy, viktar.asadchy@aalto.fi

Sci. Adv. **12**, eaed2916 (2026)
DOI: 10.1126/sciadv.aed2916

This PDF file includes:

Sections S1 to S7
Figs. S1 to S4
References

S1. Transient variation of refractive index for laser-induced plasma

In plasma, the refractive index is closely associated with the electron density after laser ionization. To calculate the electron density in air under laser ionization, we will introduce the Ammosov-Delone-Krainov (ADK) model in calculating the ionization rates and the corresponding electron density (*67,68*). The ADK model for ionization of atoms is used to describe the tunneling ionization of an electron through a suppressed potential barrier that is combined with the atomic field and the external field. The molecular ADK (MO-ADK) model is an extension for calculating the ionization rates for molecules. In the molecular frame, the wave function of the valence electron in the diatomic molecule can be expressed as:

$$\Psi^m(\mathbf{r}) = \sum_l C_l F_l(r) Y_{lm}(\mathbf{r}), \tag{S1}$$

where m is the projection of orbital angular momentum along the molecular axis, C_i is the normalized coefficient, $F_i(r)$ is for the asymptotic wave function factor that can be expressed as $F_l(r \longrightarrow \infty) \approx r^{Z_c/\kappa-1}e^{-\kappa r}$, with Z_c being the effective Coulomb charge, $\kappa = \sqrt{2I_p}$ and I_p is the ionization potential, $Y_{lm}(\mathbf{r})$ is the spherical harmonic function. Here, the atomic unit is applied, that is $m_e = \hbar = e = 1$.

By projecting the spherical harmonic along the direction of the laser field polarization and substituting the other terms in Eq. S1, the molecular wave function for tunneling ionization can be written as

$$\Psi^m(\mathbf{r}) \simeq B(m) r^{Z_c/\kappa-1} e^{-\kappa r} 2^{|m|}|m|! \sin^{|m|}\theta \frac{e^{-im\phi}}{\sqrt{2\pi}}, \tag{S2}$$

with

$$B(m) = \sum_l C_{lm}(-1)^m \sqrt{\frac{(2l+1)(l+|m|)!}{2(l-|m|)!}}, \tag{S3}$$

where the parameter C_{lm} is determined by the valence electron wave function of the molecule in the asymptotic region and can be found in Ref. (*41*).

Then, by using the method in deriving the ADK model (*67,68*), the ionization rate of a diatomic molecule under a static electric field F can therefore be written as

$$\Gamma(F) = \frac{B^2(m)}{2^{|m|}|m|!} \frac{1}{\kappa^{2Z_c/\kappa-1}} \left(\frac{2\kappa^3}{F}\right)^{2Z_c/\kappa-|m|-1} e^{-2\kappa^3/3F}. \tag{S4}$$

Here, the field strength $F(t)$ is dependent on the time delay t within the pulse duration, therefore the ionization rate $\Gamma(F)$ can also be expressed as a function of t:

$$\Gamma(t) = \frac{B^2(m)}{2^{|m|}|m|!} \frac{1}{\kappa^{2Z_c/\kappa - 1}} \left(\frac{2\kappa^3}{F(t)}\right)^{2Z_c/\kappa - |m| - 1} e^{-2\kappa^3/3F(t)}. \tag{S5}$$

By considering the depletion of the ground state, the time-dependent electron density $\rho_e(t)$ follows:

$$\frac{d\rho_e(t)}{dt} = \Gamma(t)\left[1 - \frac{\rho_e(t)}{\rho_0}\right], \tag{S6}$$

where ρ_0 is the ambient gas density in air that is about $2.447 \times 10^{19}\ \mathrm{cm}^{-3}$ by assuming that the molecules are nitrogen molecules, and $\Gamma(t)$ denotes the static tunneling ionization rate of Eq. (S5). This yields the instantaneous ionization probability $P(t) = \rho_e(t)/\rho_0$. Due to the exponential relationship between the field strength F and the static ionization rate, most ionization occurs around the sub-cycle peaks in the center of the pulse envelope. The buildup time of the cumulative ionization probability is shorter than the pulse duration of the laser pulse, which is shorter than the ps-scaled THz pulse. It results in a step-like transition of $\rho_e(t)$ as shown in FIG. 3(a) in the main text.

Next, we turn to how to calculate the refractive index of plasma at the THz frequency range during the ionization with a simple model (*33, 45*). Considering an electron with a displacement $\mathbf{x}$ in plasma under the action of THz waves $\mathbf{E}$, the motion of electrons can be written as:

$$m_e \frac{d^2\mathbf{x}}{dt^2} + m_e \gamma \frac{d\mathbf{x}}{dt} = -e\mathbf{E}, \tag{S7}$$

where γ denotes the collision damping rate determined by the velocity and the collision cross-section of electrons, m_e and e denote the mass and charge of the electron. Given the incident THz wave interacting with the electrons in plasma, the electrons are moved only along the direction of laser polarization. Since the plasma frequency of laser-prepared plasma is located in the THz frequency band and the screening effect of the electrons in plasma, it will introduce a local field correction on the localized THz field, which can be written as (*43*)

$$E_{\mathrm{loc}} = E - \frac{P}{2\varepsilon_0} \tag{S8}$$

where E_{loc} is the local field modified THz field, and P is the polarization term. Here, we assumed a cylindrical transverse structure of plasma that introduces the factor of 2 in Eq. S8. This term

introduces the structural resonance that causes the Lorentzian dispersion. With the expression $dP/dt = -e\rho_e v$, the relative dielectric constant can be written as

$$\varepsilon_{\mathrm{pl}}(\omega, t) = 1 - \frac{\omega_{\mathrm{p}}(t)^2}{\omega^2 - \omega_{\mathrm{p}}(t)^2/2 + i\gamma\omega} \tag{S9}$$

where $\omega_{\mathrm{p}}(t) = \sqrt{\rho_e(t)e^2/\varepsilon_0 m_e}$ is the plasma frequency. The real part and imaginary part of the time-dependent refractive index can therefore be written as:

$$\begin{cases} n_{\mathrm{p,r}}(\omega, t) = \mathfrak{Re}\left[\sqrt{\varepsilon_{\mathrm{p}}(\omega, t)}\right], \\ n_{\mathrm{p,i}}(\omega, t) = \mathfrak{Im}\left[\sqrt{\varepsilon_{\mathrm{p}}(\omega, t)}\right]. \end{cases} \tag{S10}$$

S2. Amplitude variation at the time boundary

As discussed above, the laser-ionized plasma will generate free electrons that abruptly change the refractive index of the incident THz waves, which can be viewed as a plasma time boundary.

At the plasma time boundary, the incident THz wave undergoes a frequency shift from the initial frequency ω_0 to a new frequency ω'. Let $A_{\mathrm{pre}}(\omega_0, t')$ denote the Fourier-transformed THz amplitude before interacting with plasma, and $A_{\mathrm{post}}(\omega', t)$ the amplitude after, at the time delay t. The shifted frequency ω' satisfies the momentum conversion relation $\omega' = \omega_0/n_{\mathrm{p,r}}(\omega', t)$, where $n_{\mathrm{p,r}}$ is the real part of the plasma refractive index (with the imaginary part denoted as $n_{\mathrm{p,i}}$, as defined in Eq. S10). t and t' denote the temporal endpoints of the incident THz waves, spanning the picosecond-scale duration of the THz pulse. Thus, the rapid change in the real part of the refractive index between these instants (from t' to t) induces the observed frequency shift.

In the experiment, the THz beam is focused to a spot size of approximately 1 mm^2, substantially larger than the laser ionization region that spans only ~0.01 mm^2 (before plasma expansion). Consequently, only a fraction of the incident THz field interacts with the plasma boundaries and undergoes a frequency shift. The portion of the THz wave that interacts with the plasma is denoted as $k(\omega_0, t)$.

The term $A_{\mathrm{post}}(\omega', t)$ can be expressed by using the incident THz amplitude spectral $A_{\mathrm{pre}}(\omega_0)$:

$$A_{\mathrm{post}}(\omega', t) = k(\omega_0, t)A_{\mathrm{pre}}(\omega_0, t')|T(\omega', t)|e^{-\alpha(\omega', t)L}, \tag{S11}$$

with L being the propagation length of the THz waves inside the plasma, the absorption coefficient

$$\alpha(\omega', t) = 2\pi n_{\mathrm{p,i}}(\omega', t)/\lambda(\omega'), \tag{S12}$$

and the transmission coefficient for the forward wave is (*3*)

$$T(\omega', t) = \frac{Z_{\rm p}(\omega', t) + Z_{\rm air}}{2Z_{\rm p}(\omega', t)} \simeq \frac{n_{\rm p}(\omega', t) + n_{\rm air}}{2n_{\rm air}} = \frac{n_{\rm p}(\omega', t) + 1}{2}, \tag{S13}$$

where $Z_i = \sqrt{\mu_i/\varepsilon_i}$ (with i = air or p) and $\mu_i \approx \mu_0$. $T(\omega', t)$ is the transmission coefficient defined for the electric displacement.

For a given frequency ω, the total received THz amplitude can be obtained from two parts, one from the interaction part and the other from the non-interaction part. Meanwhile, we assume that these amplitudes are in-phase. Thus, we can obtain:

$$A(\omega, t) = \sum_j A_{\rm post}(\omega, t) + [1 - k(\omega, t)]A_{\rm pre}(\omega, t'). \tag{S14}$$

With j, all the possible frequencies are converted from the original frequencies. Therefore, the relative THz spectral variation can be expressed as

$$\frac{\Delta A}{A}(\omega, t) = \frac{A(\omega, t) - A(\omega, t')}{A(\omega, t')}. \tag{S15}$$

Since the pulse duration of THz waves is approximately on the order of picoseconds, longer than the femtosecond laser pulse, the pre-interaction THz amplitude can be written as the incident THz amplitude, i.e., $A(\omega, t') \equiv A_{\rm pre}(\omega)$.

By substituting Eq. S11 and Eq. S14 into Eq. S15, and considering only one converted frequency satisfies the momentum conversion, that is $j = 1$, now we can obtain a formula for the spectral amplitude variation:

$$\frac{\Delta A}{A}(\omega) = \frac{k(\omega_{pl,r})A_{\rm pre}(\omega_{pl,r})|T(\omega)|e^{-\alpha(\omega)L} - k(\omega)A_{\rm pre}(\omega)}{A_{\rm pre}(\omega)}. \tag{S16}$$

Here, assumed that the interaction part of the frequency-shift is frequency independent, that is, the interaction parameter $k(\omega)$ reduces to a frequency-independent constant k, then Eq. S16 can be rewritten in a relatively simple form:

$$\frac{\Delta A}{A}(\omega) = k\left[\frac{A_{\rm pre}(\omega_{pl,r})|T(\omega)|e^{-\alpha(\omega)L} - A_{\rm pre}(\omega)}{A_{\rm pre}(\omega)}\right]. \tag{S17}$$

This formula is applicable for the frequency conversion from the original frequency to another. For a medium with a Lorentzian dispersive relationship, such as the laser-induced plasma, the original

frequency of the THz wave may have more than one choice of frequency conversion because the refractive index varies greatly in the frequency range of the resonant frequency, from greater than 1 to less than 1.

This can introduce the bi-stable structure and requires a modification of the parameters $|T|$. For the bi-stable frequency conversion shown in Fig. 2(D) of the main text, the frequency will shift to the two ends, with the maximum shift corresponding to the original frequency, whereas the middle frequencies are usually unstable and can be neglected for the lossless condition (*30*). Following the similar procedure in treating a lossless dispersive medium, the transmission coefficients T for this degenerate condition can be further expressed by adding an additional term (*30*):

$$\begin{cases} T_1(\omega_1') = \dfrac{n_{\mathrm{pl}}(\omega_1') + 1}{2} \dfrac{\varepsilon_{\mathrm{pl}}(\omega_2') - 1}{\varepsilon_{\mathrm{pl}}(\omega_2') - \varepsilon_{\mathrm{pl}}(\omega_1')}, \\ T_1(\omega_2') = \dfrac{n_{\mathrm{pl}}(\omega_2') + 1}{2} \dfrac{1 - \varepsilon_{\mathrm{pl}}(\omega_1')}{\varepsilon_{\mathrm{pl}}(\omega_2') - \varepsilon_{\mathrm{pl}}(\omega_1')}, \end{cases} \tag{S18}$$

where the frequencies ω_i' with $i = 1, 2$ satisfy the equation $\omega_0 = \omega_1' n_{\mathrm{pl},r}(\omega_1') = \omega_2' n_{\mathrm{pl},r}(\omega_2')$. The formulas in Eq. S18 can be viewed as the expression in Eq. S13 times an additional term.

The MO-ADK model employed here assumes tunneling ionization where electrons are liberated with zero average initial velocity (cold start). This corresponds to the CS (collapse-source) model (*48*) where the source term $s(t) = 0$ during the plasma generation, and the boundary conditions follow the passive increase case of Galiffi *et al.* (*27*): the electric displacement $\mathbf{D}$ is conserved, while the electromagnetic energy decreases as it converts into electron kinetic energy. As shown in Fig. 3(A), the transmittance of THz waves for the time boundary and the space boundary are 96.8% and 64.4%, respectively, consistent with the energy reduction results in Ref. (*27, 48*). The electron-ion recombination time (∼10 ns) far exceeds our experimental observation window (~ps), ensuring we only encounter the plasma creation without needing the momentum-removal source terms required for the collapse part (*48*). These conditions are physically equivalent to the continuity of $\mathbf{P}$ and $\dot{\mathbf{P}}$ assumed in Solís *et al.* (*30*), validating the use of Eq. S18 for transmission coefficients.

Based on the coefficients $|T|$, we can calculate the spectral variations of the transmitted THz waves from the experimental results, as shown in FIG. 2(c) in the main text. It is worth noting that the expression of Eq. S18 originates from the lossless condition, which neglects the frequency conversion to the unstable solutions of the bi-stable frequency conversion. This leads to a zero-valued region in $\Delta A/A(\omega)$ because A_{post} cannot be converted into the unstable frequency range.

S3. Amplitude variation at the space boundary

When the plasma is formed before the transmission of THz waves, this plasma will only act as a spatial boundary for the input THz waves. Then, the space boundary is mainly induced by two reasons for the relative spectral variations: one is the spatial reflection of the incident THz wave, governed by

$$R(\omega)^2 = \frac{\left[(n_r(\omega) - 1)^2 + n_i(\omega)^2\right]}{\left[(n_r(\omega) + 1)^2 + n_i(\omega)^2\right]}, \tag{S19}$$

the other is the plasma absorption of the index $e^{-\alpha(\omega_0)L}$. Based on the two reasons, the relative spectral variation can therefore be expressed as:

$$\frac{\Delta A}{A}(\omega) = k' \left\{\left[1 - R(\omega)^2\right]^2 e^{-\alpha(\omega)L'} - 1\right\}. \tag{S20}$$

Here, the interaction parameter k' is assumed to be frequency-independent, and L' is the propagation length of the THz wave inside the plasma. It is worth noting that due to the plasma expansion after the generation of plasma, the values of the parameters k' and L' at the space boundary can be larger than the values of the time boundary.

S4. Photon acceleration and time-boundary effect

When an incident electromagnetic wave encounters the plasma time boundary, two mechanisms may contribute to the frequency shifts: the time-boundary effects (flash ionization) (*34*) and photon acceleration (*35*). The principle of photon acceleration treats the ionization front of the plasma as a moving mirror, causing a Doppler shift of the incident electromagnetic wave (*35*). This mechanism predicts only unidirectional blue-shift. In contrast, the time-boundary effect arises from the instantaneous change in plasma permittivity, which can produce bidirectional frequency splitting through the Drude-Lorentz resonance model (see Fig. 1C and Eq. 4 in the main text). This is evident in our experimental results, which show a prominent red-shifted branch that can not be explained by photon acceleration alone.

For the low-frequency wing of the bidirectional frequency shifts, the peak positions undergo systematic red-shifts with increasing pump intensity. As shown in Fig. S1, when the pump energy rises from 1.0 to 1.4 mJ, the peak of amplitude variation in the low-frequency wing moves progressively from 0.21 THz to a lower frequency 0.13 THz ($\Delta f \approx -0.08$ THz), consistent with increased

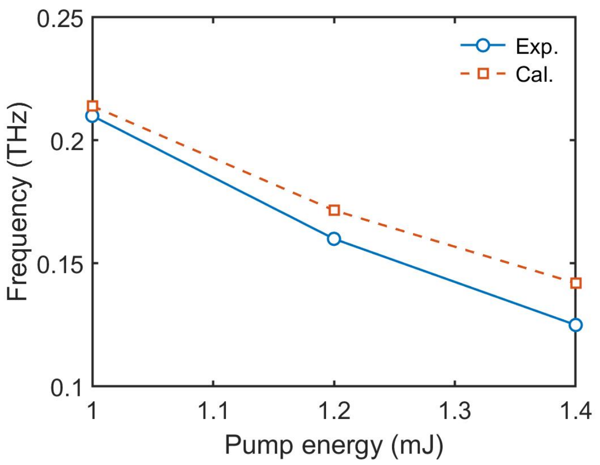

Figure S1: Frequency peaks in amplitude variation in the low frequency range as a function of laser energy.

refractive index changes driving larger frequency shifts (red-shifts in the low-frequency wing). This trend aligns with our Drude-Lorentz model (red dashed lines), providing direct validation of pump-intensity-dependent frequency shifts of the peaks in amplitude variation that support the time boundary interpretation.

Furthermore, previous studies show that photon acceleration dominates the frequency up-shifts before the focal point in loose-focus systems (*69*) or with chirped pulses where the ionization front velocity differs greatly from the speed of light c (*38, 70*). However, our experiment uses tight focusing (7.62 cm focal length) and Fourier-transform-limited pulses, localizing the plasma boundary to ~ 2 mm around the focus—longer than the most sub-mm-scaled THz wavelengths to avoid edge effects (*71*).

Therefore, we can safely neglect photon acceleration in our analysis, and the observed effects are dominated by the time boundary effects.

S5. Analysis of the walk-off between IR and THz waves

The pump beam propagates at the speed of light in air, while the propagation of the THz wave is changed by the plasma dispersion, experiencing a dramatic change in refractive index near the resonance frequency. Therefore, it is needed to analyze the walk-off between the IR pump beam and THz waves.

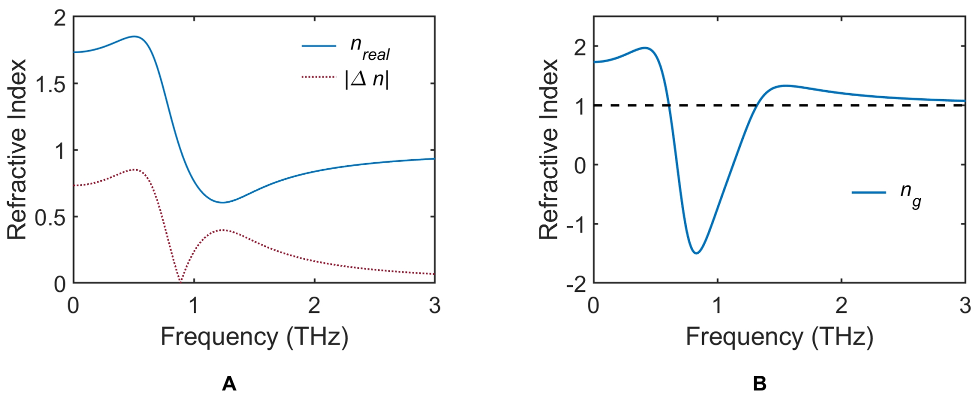

Figure S2: (**A**) Real part of the plasma refractive index (blue solid line) and the refractive index change between plasma and air (red dashed line). (**B**) Calculated group refractive index at a laser intensity of 1.7×10^{14} W/cm^2.

For the frequencies far from the structural resonance, there is a difference in the phase velocity between the THz wave and the pump infrared light. Based on Eq. (S10), the difference between the refractive index of the THz wave at frequency ω in plasma and that of light in air can be written as

$$\Delta n = |1 - n_{\mathrm{p,r}}(\omega)| = \Re\mathrm{e}\left|1 - \sqrt{1 - \frac{\omega_p^2}{\omega^2 - \omega_p^2/2 + i\gamma\omega}}\right|. \tag{S21}$$

Figure S2(A) shows the calculation result of Δn. The walk-off length for 2π phase shift (an optical cycle) can be calculated by:

$$L = \frac{2\pi c}{\omega \Delta n}. \tag{S22}$$

The result substantially exceed our plasma length of 2 mm, rendering the induced time delay negligible to the incident THz pulse.

For the frequencies near the structural resonance, the phase velocity fails to describe the actual pulse propagation due to the strong dispersion. Typically, the group velocity $v_g = c/n_g$ is applied to estimate the walk-off of pulse envelop. However, as shown in Fig. S2(B), $n_g < 1$ (or even negative) near the resonance, suggesting nonphysical superluminal propagation of THz wave at $v_g > c$. It is well known that the concept of group velocity applies only in transparent low-dispersive regions and it loses physical meaning in the absorbing regions (*72*). Therefore, in the resonance region, a more appropriate treatment is to use the signal velocity v_s following the Sommerfeld-Brillouin theory (*73*) instead of the group velocity, which satisfies causality with $v_s \approx c$ (*74*). The temporal

walk-off between the IR and THz waves is thus determined by the Sommerfeld precursor wave propagating at $v_s \approx c$, maintaining the temporal overlap. Figure 3(A) in the main text also shows evidence that the wavefronts of the incident, time-boundary, and space-boundary transmitted THz pulses are temporally aligned, confirming that the THz wavefront co-propagates with the IR pulse under the three scenarios.

In conclusion, the THz wave co-propagates nearly synchronously with the IR pulse, ensuring the minimal walk-off induced by the plasma dispersion.

S6. Mode profiles and propagation characteristics of low-frequency THz waves in the plasma filament

In this section, we clarify the mode profiles and propagation characteristics of THz waves in the plasma filament. The high-frequency (blue-shifted) THz components are frequencies above the resonance frequency and thus propagate through the plasma with low loss, behaving like ordinary transmitted waves in a dielectric medium. For the lower frequency branch of THz waves, it propagates as a guided mode within the plasma, not as a surface wave.

To clarify the low-frequency branch properties, we performed simulations of THz propagation in a uniform cylindrical plasma with radius 50 μm. The permittivity is calculated from the Drude-Lorentz model Eq. (S9). The eigenmodes of the THz wave inside and outside the plasma filament are shown in Fig. S3 and S4, respectively. At 0.15 THz (below the resonance), the field is confined within the ring-shaped region of the plasma cylinder [Fig. S3(E)]. It can propagate and couple out effectively [Fig. S4(E)]. At extremely low frequencies (*e.g.*, 0.03 THz), confinement weakens [Fig. S3(A)] and energy radiates outward [Fig. S4(A)].

Real plasma has radial density gradients. Simulations with the density gradient profile show that low frequencies are confined in rings of specific thickness, with skin depths exceeding 1 mm (*75*). The eigenmode distribution confirms transmission occurs within plasma rings at different radial distances (*76*).

Extremely low frequency waves concentrate on the plasma ring's outer edge, where energy leakage reduces the transmission efficiency, and large divergence angles make the collection by off-axis parabolic mirrors (NA ~ 0.31) inefficient. These two factors explain the suppression of

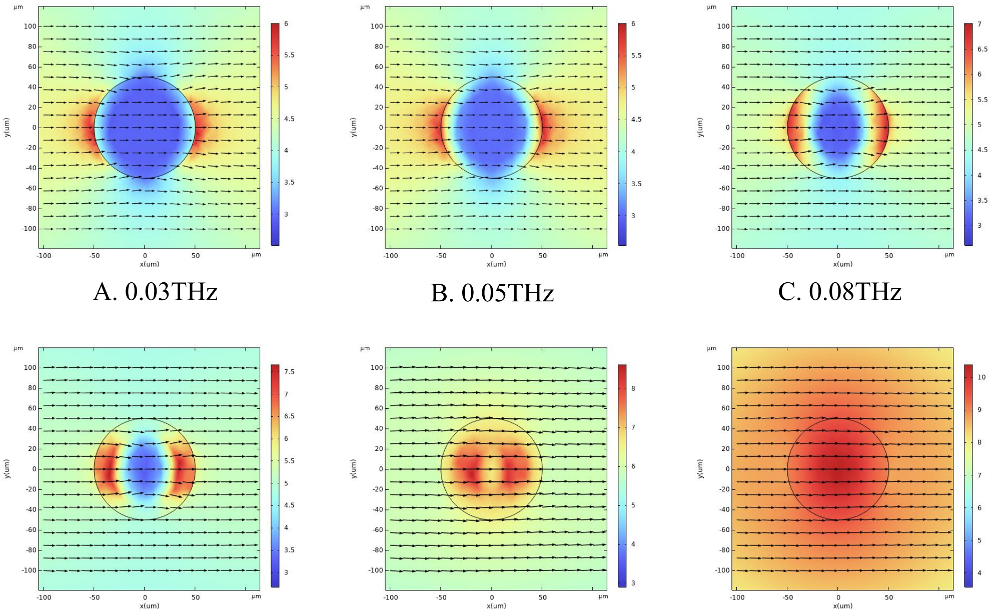

A. 0.03THz B. 0.05THz C. 0.08THz

D. 0.1THz E. 0.15THz F. 0.25THz

Figure S3: THz eigenmode profile inside plasma filament. Poynting vector (color) and electric vector (arrows) for frequencies from 0.03 to 0.25 THz. The plasma boundary is indicated by black [illegible]

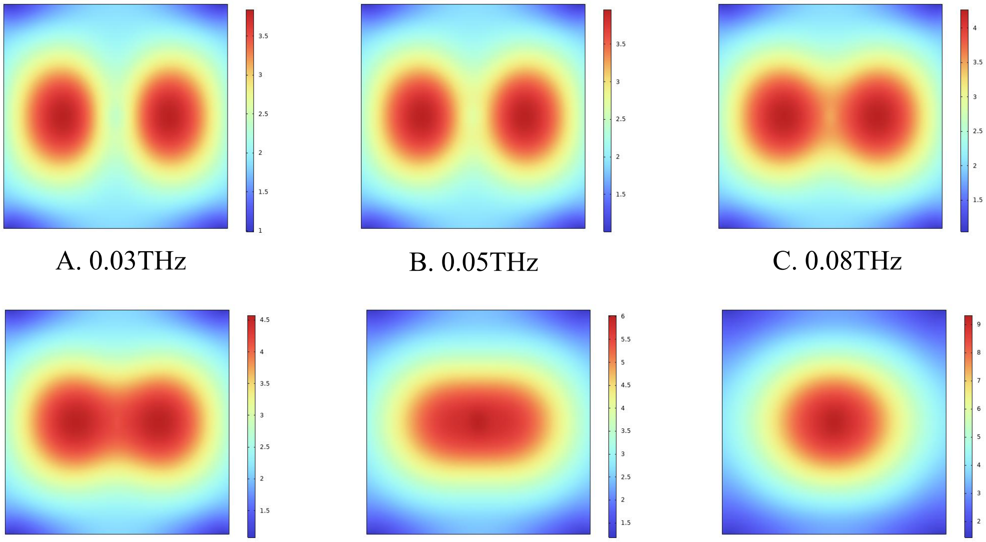

A. 0.03THz B. 0.05THz C. 0.08THz

D. 0.1THz E. 0.15THz F. 0.25THz

Figure S4: Output THz eigenmodes at plasma filament exit.

extremely low-frequency components (0.1 THz) observed in Fig. 4C of the main text.

Therefore, both frequency branches can propagate in a plasma filament, but the red-shifted branch experiences intrinsic confinement and collection losses at extremely low frequencies. This confirms that the low-frequency signals originate from guided plasma modes, not the surface waves, validating the time-boundary interpretation.

S7. Comparison with other time-varying platforms

Following the analysis framework in Ref. (*28*), the strength of frequency conversion for different time-varying platforms can be calculated and compared by using

$$\Delta f / f = -\Delta n / (n_0 + \Delta n), \tag{S23}$$

where Δn is the refractive index change and n_0 is the initial refractive index.

Based on the data presented in Figure 2C, we can calculate the maximum value of the refractive index changes:

(1) for 0.5 THz: $\Delta n = +0.8 \rightarrow \Delta f / f = -44\%$ (red-shift of about 0.22 THz);

(2) for 1.1 THz: $\Delta n = -0.4 \rightarrow \Delta f / f = +67\%$ (blue-shift of about 0.40 THz).

As shown in Table 1, our air-plasma platform achieves the largest strength of Δn in the THz regime. These results demonstrate that plasma uniquely combines strong frequency conversion with low loss in the THz frequency range, as well as the high damage threshold and spatial reconfigurability. It makes plasma a suitable platform for THz time-varying photonics.

REFERENCES

1. J. T. Mendonça, *Theory of Photon Acceleration* (Institute of Physics Publishing, 2001).

2. B. W. Plansinis, W. R. Donaldson, G. P. Agrawal, What is the temporal analog of reflection and refraction of optical beams? *Phys. Rev. Lett.* **115**, 183901 (2015).

3. E. Galiffi, R. Tirole, S. Yin, H. Li, S. Vezzoli, P. A. Huidobro, M. G. Silveirinha, R. Sapienza, A. Alù, J. B. Pendry, Photonics of time-varying media. *Adv. Photonics* **4**, 014002 (2022).

4. L. Bar-Hillel, A. Dikopoltsev, A. Kam, Y. Sharabi, O. Segal, E. Lustig, M. Segev, Time refraction and time reflection above critical angle for total internal reflection. *Phys. Rev. Lett.* **132**, 263802 (2024).

5. M. M. Asgari, P. Garg, X. Wang, M. S. Mirmoosa, C. Rockstuhl, V. Asadchy, Theory and applications of photonic time crystals: A tutorial. *Adv. Opt. Photonics* **16**, 958–1063 (2024).

6. Z. Hayran, F. Monticone, A resonant tone for photonic time crystals. *Nat. Photonics* **19**, 126–128 (2025).

7. M. Lyubarov, Y. Lumer, A. Dikopoltsev, E. Lustig, Y. Sharabi, M. Segev, Amplified emission and lasing in photonic time crystals. *Science* **377**, 425–428 (2022).

8. X. Wang, P. Garg, M. S. Mirmoosa, A. G. Lamprianidis, C. Rockstuhl, V. S. Asadchy, Expanding momentum bandgaps in photonic time crystals through resonances. *Nat. Photonics* **19**, 149–155 (2025).

9. N. Konforty, M.-I. Cohen, O. Segal, Y. Plotnik, V. M. Shalaev, M. Segev, Second harmonic generation and nonlinear frequency conversion in photonic time-crystals. *Light Sci. Appl.* **14**, 152 (2025).

10. E. Galiffi, Y.-T. Wang, Z. Lim, J. B. Pendry, A. Alù, P. A. Huidobro, Wood anomalies and surface-wave excitation with a time grating. *Phys. Rev. Lett.* **125**, 127403 (2020).

11. Y. Sharabi, E. Lustig, M. Segev, Disordered photonic time crystals. *Phys. Rev. Lett.* **126**, 163902 (2021).

12. J. Kim, D. Lee, S. Yu, N. Park, Unidirectional scattering with spatial homogeneity using correlated photonic time disorder. *Nat. Phys.* **19**, 726–732 (2023).

13. Z. Dong, X. Chen, L. Yuan, Extremely narrow band in moiré photonic time crystal. *Phys. Rev. Lett.* **135**, 033803 (2025).

14. Y. Sharabi, A. Dikopoltsev, E. Lustig, Y. Lumer, M. Segev, Spatiotemporal photonic crystals. *Optica* **9**, 585–592 (2022).

15. X. Wang, M. S. Mirmoosa, V. S. Asadchy, C. Rockstuhl, S. Fan, S. A. Tretyakov, Metasurface-based realization of photonic time crystals. *Sci. Adv.* **9**, eadg7541 (2023).

16. W. Jaffray, S. Stengel, F. Biancalana, C. Fruhling, M. Ozlu, M. Scalora, A. Boltasseva, V. Shalaev, M. Ferrera, Spatio-spectral optical fission in time-varying subwavelength layers. *Nat. Photonics* **19**, 558–566 (2025).

17. H. Moussa, G. Xu, S. Yin, E. Galiffi, Y. Ra'di, A. Alú, Observation of temporal reflection and broadband frequency translation at photonic time interfaces. *Nat. Phys.* **19**, 863–868 (2023).

18. T. Jones, A. Kildishev, M. Segev, D. Peroulis, Time-reflection of microwaves by a fast optically-controlled time-boundary. *Nat. Commun.* **15**, 6786 (2024).

19. A. Nishida, N. Yugami, T. Higashiguchi, T. Otsuka, F. Suzuki, M. Nakata, Y. Sentoku, R. Kodama, Experimental observation of frequency up-conversion by flash ionization. *Appl. Phys. Lett.* **101**, 161118 (2012).

20. F. Miyamaru, C. Mizuo, T. Nakanishi, Y. Nakata, K. Hasebe, S. Nagase, Y. Matsubara, Y. Goto, J. Pérez-Urquizo, J. Madéo, K. M. Dani, Ultrafast frequency-shift dynamics at temporal boundary induced by structural-dispersion switching of waveguides. *Phys. Rev. Lett.* **127**, 053902 (2021).

21. M. Z. Alam, I. D. Leon, R. W. Boyd, Large optical nonlinearity of indium tin oxide in its epsilon-near-zero region. *Science* **352**, 795–797 (2016).

22. R. Tirole, S. Vezzoli, E. Galiffi, I. Robertson, D. Maurice, B. Tilmann, S. A. Maier, J. B. Pendry, R. Sapienza, Double-slit time diffraction at optical frequencies. *Nat. Phys.* **19**, 999–1002 (2023).

23. J. Bohn, T. S. Luk, S. Horsley, E. Hendry, Spatiotemporal refraction of light in an epsilon-near-zero indium tin oxide layer: Frequency shifting effects arising from interfaces. *Optica* **8**, 1532–1537 (2021).

24. E. Lustig, O. Segal, S. Saha, E. Bordo, S. N. Chowdhury, Y. Sharabi, A. Fleischer, A. Boltasseva, O. Cohen, V. M. Shalaev, M. Segev, Time-refraction optics with single cycle modulation. *Nanophotonics* **12**, 2221–2230 (2023).

25. Y. Lu, X. Zhang, H. Qiu, L. Niu, X. Chen, Q. Xu, W. Zhang, S. Zhang, J. Han, Ultrafast temporal modulation of terahertz generation at an optically pumped ITO interface. *Optica* **12**, 1035–1043 (2025).

26. F. Morgenthaler, Velocity modulation of electromagnetic waves. *IRE Trans. Microw. Theory Tech.* **6**, 167–172 (1958).

27. E. Galiffi, D. M. Solís, S. Yin, N. Engheta, A. Alù, Electrodynamics of photonic temporal interfaces. *Light Sci. Appl.* **14**, 338 (2025).

28. Y. Zhou, M. Z. Alam, M. Karimi, J. Upham, O. Reshef, C. Liu, A. E. Willner, R. W. Boyd, Broadband frequency translation through time refraction in an epsilon-near-zero material. *Nat. Commun.* **11**, 2180 (2020).

29. K. Qu, Q. Jia, M. R. Edwards, N. J. Fisch, Theory of electromagnetic wave frequency upconversion in dynamic media. *Phys. Rev. E* **98**, 023202 (2018).

30. D. M. Solís, R. Kastner, N. Engheta, Time-varying materials in the presence of dispersion: Plane-wave propagation in a Lorentzian medium with temporal discontinuity. *Photonics Res.* **9**, 1842–1853 (2021).

31. C. Rizza, M. A. Vincenti, G. Castaldi, A. Contestabile, V. Galdi, M. Scalora, Harnessing the natural resonances of time-varying dispersive interfaces. *Phys. Rev. Lett.* **133**, 186902 (2024).

32. P. Garg, E. Almpanis, L. Zimmer, J. D. Fischbach, X. Wang, M. S. Mirmoosa, M. Nyman, N. Stefanou, N. Papanikolaou, V. Asadchy, C. Rockstuhl, Photonic time crystals assisted by quasi-bound states in the continuum. arXiv:2507.15644 [physics.optics] (2025).

33. Z. Zheng, Y. Huang, Q. Guo, C. Meng, Z. Lu, X. Wang, J. Zhao, C. Meng, D. Zhang, J. Yuan, Z. Zhao, Filament characterization via resonance absorption of terahertz wave. *Phys. Plasmas* **24**, 103303 (2017).

34. S. P. Kuo, Frequency up-conversion of microwave pulse in a rapidly growing plasma. *Phys. Rev. Lett.* **65**, 1000–1003 (1990).

35. J. M. Dias, C. Stenz, N. Lopes, X. Badiche, F. Blasco, A. Dos Santos, L. Oliveira e Silva, A. Mysyrowicz, A. Antonetti, J. T. Mendonça, Experimental evidence of photon acceleration of ultrashort laser pulses in relativistic ionization fronts. *Phys. Rev. Lett.* **78**, 4773–4776 (1997).

36. I. Geltner, Y. Avitzour, S. Suckewer, Picosecond pulse frequency upshifting by rapid free-carrier creation in ZnSe. *Appl. Phys. Lett.* **81**, 226–228 (2002).

37. M. R. Edwards, K. Qu, Q. Jia, J. M. Mikhailova, N. J. Fisch, Cascaded chirped photon acceleration for efficient frequency conversion. *Phys. Plasmas* **25**, 053102 (2018).

38. A. J. Howard, D. Turnbull, A. S. Davies, P. Franke, D. H. Froula, J. P. Palastro, Photon acceleration in a flying focus. *Phys. Rev. Lett.* **123**, 124801 (2019).

39. R. T. Sandberg, A. G. R. Thomas, Photon acceleration from optical to XUV. *Phys. Rev. Lett.* **130**, 085001 (2023).

40. X. Xu, Y. Huang, Z. Zhang, J. Liu, J. Lou, M. Gao, S. Wu, G. Fang, Z. Zhao, Y. Chen, Z. Sheng, C. Chang, Laser-chirp controlled terahertz wave generation from air plasma. *Chin. Phys. Lett.* **40**, 045201 (2023).

41. X. M. Tong, Z. X. Zhao, C. D. Lin, Theory of molecular tunneling ionization. *Phys. Rev. A* **66**, 033402 (2002).

42. N. Shlomo, E. Frumker, In situ characterization of laser-induced strong field ionization phenomena. *Light Sci. Appl.* **14**, 166 (2025).

43. Z. Mics, F. Kadlec, P. Kužel, P. Jungwirth, S. E. Bradforth, V. A. Apkarian, Nonresonant ionization of oxygen molecules by femtosecond pulses: Plasma dynamics studied by time-resolved terahertz spectroscopy. *J. Chem. Phys.* **123**, 104310 (2005).

44. Z. Mics, P. Kužel, P. Jungwirth, S. E. Bradforth, Photoionization of atmospheric gases studied by time-resolved terahertz spectroscopy. *Chem. Phys. Lett.* **465**, 20–24 (2008).

45. Y. Huang, Z. Xiang, X. Xu, J. Zhao, J. Liu, R. Wang, Z. Zhang, Z. Lü, D. Zhang, C. Chang, J. Yuan, Z. Zhao, Localized-plasma-assisted rotational transitions in the terahertz region. *Phys. Rev. A* **103**, 033109 (2021).

46. S. P. Kuo, A. Ren, G. Schmidt, Frequency downshift in rapidly ionizing media. *Phys. Rev. E* **49**, 3310–3315 (1994).

47. S. Undurti, H. Xu, X. Wang, A. Noor, W. Wallace, N. Douguet, A. Bray, I. Ivanov, K. Bartschat, A. Kheifets, R. Sang, I. Litvinyuk, Attosecond angular streaking and tunnelling time in atomic hydrogen. *Nature* **568**, 75–77 (2019).

48. H. Mehrpour Bernety, D. K. Kalluri, M. A. Cappelli, Proper treatment of energy and momentum in time-modulated plasmas. *Phys. Rev. E* **113**, 025204 (2026).

49. X.-K. Wang, J.-S. Ye, W.-F. Sun, P. Han, L. Hou, Y. Zhang, Terahertz near-field microscopy based on an air-plasma dynamic aperture. *Light Sci. Appl.* **11**, 129 (2022).

50. J. Zhao, F. Zhu, Y. Han, Q. Wang, L. Lao, X. Li, Y. Peng, Y. Zhu, Light-guiding-light-based temporal integration of broadband terahertz pulses in air. *APL Photonics* **8**, 106107 (2023).

51. G. Xu, H. Xing, D. Lu, J. Fan, Z. Xue, P. P. Shum, L. Cong, Linear terahertz frequency conversion in a temporal-boundary metasurface. *Laser Photonics Rev.* **18**, 2301294 (2024).

52. I. Ahmad, L. Bergé, Z. Major, F. Krausz, S. Karsch, S. A. Trushin, Redshift of few-cycle infrared pulses in the filamentation regime. *New J. Phys.* **13**, 093005 (2011).

53. H. Kim, C. M. Kim, K. H. Pae, K. T. Kim, Isolated attosecond pulses generated from a relativistic plasma mirror via noncollinear gating. *Phys. Rev. Res.* **7**, 013216 (2025).

54. X. Qu, Y. Huang, B. Zhou, M. Gao, J. Lou, Y. Feng, Z. Zhao, C. Chang, A. P. Shkurinov, J. Verboncoeur, Ultrafast plasma-based terahertz modulator. *Optica* **11**, 1478–1481 (2024).

55. J. Papeer, D. Gordon, P. Sprangle, M. Botton, A. Zigler, Temporal evolution of femtosecond laser induced plasma filament in air and N_2. *Appl. Phys. Lett.* **103**, 244102 (2013).

56. T. Kampfrath, D. O. Gericke, L. Perfetti, P. Tegeder, M. Wolf, C. Frischkorn, Long- and short-lived electrons with anomalously high collision rates in laser-ionized gases. *Phys. Rev. E* **76**, 066401 (2007).

57. Y. Schrödel, C. Hartmann, J. Zheng, T. Lang, M. Steudel, M. Rutsch, S. H. Salman, M. Kellert, M. Pergament, T. Hahn-Jose, S. Suppelt, J. H. Dörsam, A. Harth, W. P. Leemans, F. X. Kärtner, I. Hartl, M. Kupnik, C. M. Heyl, Acousto-optic modulation of gigawatt-scale laser pulses in ambient air. *Nat. Photonics* **18**, 54–59 (2024).

58. X.-B. Zhang, S.-M. Weng, H. Ai, X. Qiao, J.-K. Xue, Z.-M. Sheng, Photonic rabi oscillations in defective plasma photonic crystals. *Phys. Rev. Lett.* **135**, 015101 (2025).

59. G. Lehmann, K. H. Spatschek, Transient plasma photonic crystals for high-power lasers. *Phys. Rev. Lett.* **116**, 225002 (2016).

60. N. L. Wagner, E. A. Gibson, T. Popmintchev, I. P. Christov, M. M. Murnane, H. C. Kapteyn, Self-compression of ultrashort pulses through ionization-induced spatiotemporal reshaping. *Phys. Rev. Lett.* **93**, 173902 (2004).

61. Y. Pan, M.-I. Cohen, M. Segev, Superluminal *k*-gap solitons in nonlinear photonic time crystals. *Phys. Rev. Lett.* **130**, 233801 (2023).

62. Y. Jang, B. Oh, E. Kim, J. Rho, Bidirectional asymmetric frequency conversion in nonlinear phononic crystals. *Phys. Rev. Lett.* **135**, 036603 (2025).

63. J. Dong, S. Zhang, H. He, H. Li, J. Xu, Nonuniform wave momentum band gap in biaxial anisotropic photonic time crystals. *Phys. Rev. Lett.* **134**, 063801 (2025).

64. J. Ying, L. Liu, L. Zheng, D. Su, X. He, J. Ma, H. Xuan, D. Zhang, High-energy subcycle electron emission driven by spatiotemporally confined THz fields. *Phys. Rev. X* **15**, 021095 (2025).

65. N. Karpowicz, J. Dai, X. Lu, Y. Chen, M. Yamaguchi, H. Zhao, X.-C. Zhang, L. Zhang, C. Zhang, M. Price-Gallagher, C. Fletcher, O. Mamer, A. Lesimple, K. Johnson, Coherent heterodyne time-domain spectrometry covering the entire "terahertz gap". *Appl. Phys. Lett.* **92**, 011131 (2008).

66. M. R. Shcherbakov, S. Liu, V. Zubyuk, A. Vaskin, P. Vabishchevich, G. Keeler, T. Pertsch, T. Dolgova, I. Staude, I. Brener, A. Fedyanin, Ultrafast all-optical tuning of direct-gap semiconductor metasurfaces. *Nat. Commun.* **8**, 17 (2017).

67. B. M. Smirnov, M. I. Chibisov, The breaking up of atomic particles by an electric field and by electron collisions. *Sov. Phys. JETP* **22**, 585–592 (1966).

68. M.-V. Ammosov, N.-B. Delone, V.-P. Krainov, Tunnel ionization of complex atoms and atomic ions in electromagnetic field. *Sov. Phys. JETP* **64**, 1191–1194 (1986).

69. J. M. Dias, N. C. Lopes, L. O. Silva, G. Figueira, J. T. Mendonça, C. Stenz, F. Blasco, A. Dos Santos, A. Mysyrowicz, Photon acceleration of ultrashort laser pulses by relativistic ionization fronts. *Phys. Rev. E* **66**, 056406 (2002).

70. S. Fu, B. Groussin, Y. Liu, A. Mysyrowicz, V. Tikhonchuk, A. Houard, Steering laser-produced THz radiation in air with superluminal ionization fronts. *Phys. Rev. Lett.* **134**, 045001 (2025).

71. S. C. Wilks, J. M. Dawson, W. B. Mori, Frequency up-conversion of electromagnetic radiation with use of an overdense plasma. *Phys. Rev. Lett.* **61**, 337–340 (1988).

72. R. Loudon, The propagation of electromagnetic energy through an absorbing dielectric. *J. Phys. A: Gen. Phys.* **3**, 233–245 (1970).

73. L. Brillouin, *Wave Propagation and Group Velocity*. (Academic Press, 1960).

74. K. Oughstun, C. Balictsis, Gaussian pulse propagation in a dispersive, absorbing dielectric. *Phys. Rev. Lett.* **77**, 2210–2213 (1996).

75. J. Zhao, Y. Zhang, Z. Wang, W. Chu, B. Zeng, W. Liu, Y. Cheng, Z. Xu, Propagation of terahertz wave inside femtosecond laser filament in air. *Laser Phys. Lett.* **11**, 095302 (2014).

76. J. Zhao, W. Chu, Z. Wang, Y. Peng, C. Gong, L. Lin, Y. Zhu, W. Liu, Y. Cheng, S. Zhuang, Z. Xu, Strong spatial confinement of terahertz wave inside femtosecond laser filament. *ACS Photonics* **3**, 2338–2343 (2016).
