# Supplementary material for: Bidirectional terahertz frequency conversion via structural resonances at a plasma time boundary
Source: Sci Adv. 2026 Jul 31;12(31):eaed2916. doi: 10.1126/sciadv.aed2916 (PMC13426419; doi:10.1126/sciadv.aed2916)
Supplement: Supplementary file 2 [file sciadv.aed2916.v1.pdf]

APPLIED PHYSICS

Bidirectional terahertz frequency conversion via structural resonances at a plasma time boundary

Yindong Huang[1]*†, Bin Zhou[1,2]†, Aijun Xuan[1,3], Mingxin Gao[1,4], Jing Lou[1], Xiaomin Qu[1], Zengxiu Zhao[4], Ce Shang[5]*, Xuchen Wang[6]*, Chao Chang[1,7]*, Viktar Asadchy[8]*

Copyright © 2026 The Authors, some rights reserved; exclusive licensee American Association for the Advancement of Science. No claim to original U.S. Government Works. Distributed under a Creative Commons Attribution License 4.0 (CC BY).

A time boundary, an abrupt temporal change in material refractive index that conserves wave vector while shifting optical frequency, offers a powerful route to dynamic spectral control. Prior demonstrations of time boundaries in Drude-like materials have predominantly produced simple, unidirectional frequency shifts. Here, we experimentally realize an ultrafast time boundary for terahertz waves at an interface between air and a laser-induced plasma that exhibits a Lorentzian resonant response via a localized surface-plasmon mode. The boundary is created by converting air into a cylindrical plasma column within 100 femtoseconds, enabling strong, sub-cycle index modulation. We observe time refraction with unconventional bidirectional (simultaneous red- and blue-shifted) frequency conversion at a single boundary. A simple model that couples a tunneling-ionization description of plasma formation with Lorentzian dispersion quantitatively reproduces the measured spectra. By precisely tuning the delay between plasma creation and terahertz-wave arrival, we resolve the spectral evolution in time and directly confirm time refraction as a fundamental sub-cycle effect. These results establish Lorentzian time boundaries as an experimental platform for rich temporal light-matter interactions and lay groundwork for dynamic terahertz photonics, including temporally reconfigurable spectral elements, amplification schemes, and building blocks for photonic time crystals and spatiotemporal metamaterials.

INTRODUCTION

The refractive index of a medium governs how light propagates within it. Spatially manipulating this index using elements such as lenses, gratings, or metamaterials allows precise control over light's direction, energy, and phase. Extending this control to the time domain, an abrupt temporal change in the refractive index, termed a time boundary, conserves wave vector while shifting frequency, leading to intriguing wave phenomena like time refraction and time reflection (*1*). Unlike conventional spatial interfaces that redirect light in space, time boundaries dynamically reshape spectra in real time, providing a powerful tool for dynamic photonic control (*2*–*4*). These temporal light-matter interactions form the foundation of emerging photonic time crystals (*5*, *6*) and enable transformative applications including optical amplification (*7*–*9*), temporal optical elements (*10*–*13*), and spatiotemporally engineered photonics (*14*–*16*).

Time-boundary–induced spectral control has been demonstrated across the electromagnetic spectrum, from microwaves to the near-infrared. In the microwave regime, time reflection has been realized using electrically modulated metamaterials (*17*) and optically gated photodiodes (*18*). In the terahertz (THz) range, laser-excited ZnSe crystals (*19*) and metal-semiconductor waveguides (*20*) exhibit blue shifts via time refraction. In the near-infrared, time refraction in photoexcited transparent conducting oxides (TCOs) (*21*) has enabled time-slit interference (*22*), controlled spectral shifts of the fundamental wave (*23*, *24*), and associated THz generation (*25*). Despite these advances, both the observed phenomena and the prevailing models have largely remained limited to time reflection and time refraction with simple, unidirectional frequency conversions, an outcome of the Drude-like or effectively dispersionless character of the media typically used (*26*, *27*). Recent progress has demonstrated broadband red and blue shifts sequentially at the respective rising and falling edges in TCOs via rapid generation and relaxation of the epsilon-near-zero effect (*28*). This sequential nature with a single frequency shift at a temporal boundary is inherent to the monotonic temporal response of Drude-type systems. Theory, in contrast, predicts that time boundaries in Lorentzian media can yield qualitatively richer behavior, including the generation of multiple shifted frequencies simultaneously at a single boundary (*29*–*31*) and the creation of infinitely wide momentum bandgaps in periodically cascaded boundaries (*8*, *32*). To date, an experimental realization of time boundaries in materials with Lorentzian resonant dispersion has not been reported.

Here, we experimentally realize an ultrafast time boundary for a THz wave at an interface between air and material with Lorentzian resonant response, and we observe a time-refracted beam with bidirectional (simultaneous red- and blue-shifted) frequency conversion. The time boundary is created by converting air into a column of laser-induced plasma within ~100 fs. While plasma is intrinsically Drude like, its finite cylindrical confinement supports a localized surface-plasmon resonance (*33*), effectively imparting a Lorentzian dispersive response. Therefore, we refer to this resonance as "structural," arising from the geometry and boundary conditions of the plasma column, rather than an intrinsic material resonance. Moreover, laser-induced plasma is ideally suited for strong, rapid index modulation, providing refractive-index contrasts of ~100% on femtosecond timescales (*34*–*39*). Combining a tunneling-ionization model for plasma formation with this Lorentzian dispersion, we develop a simple

[1]Innovation Laboratory of Terahertz Biophysics, National Innovation Institute of Defense Technology, Beijing 100071, China. [2]Department of Physics, Tsinghua University, Beijing 100084, China. [3]School of Mathematics and Physics, North China Electric Power University, Beijing 102206, China. [4]College of Science, National University of Defense Technology, Changsha 410073, China. [5]Aerospace Information Research Institute, Chinese Academy of Sciences, Beijing 100094, China. [6]College of Physics and Optoelectronic Engineering, Harbin Engineering University, Harbin 150001, China. [7]School of Physics, Peking University, Beijing 100871, China. [8]Department of Electronics and Nanoengineering, Aalto University, Maarintie 8, 02150 Espoo, Finland.
*Corresponding author. Email: yindonghuang@nudt.edu.cn (Y.H.); shangce@aircas.ac.cn (C.S.); xuchen.wang@hrbeu.edu.cn (X.W.); gwyzlzssb@pku.edu.cn (C.C.); viktar.asadchy@aalto.fi (V.A.)
†These authors contributed equally to this work.

model that quantitatively reproduces the observed unconventional frequency shifts. By precisely tuning the delay between plasma creation and THz-wave arrival, we resolve the spectral evolution in time, directly confirming time refraction as a fundamental sub-cycle time-boundary effect.

RESULTS

Concept and experimental realization

As illustrated in Fig. 1A, synchronized femtosecond laser ionization establishes a transient plasma time boundary during THz wave propagation, inducing measurable spectral shifts via time refraction. The inset illustrates frequency conversion from initial frequency ω_0 to ω' across this time boundary. An abrupt change in the real part of refractive index $n_{p,r}(\omega', t)$ alters the phase velocity of light while conserving momentum, thus resulting in the frequency shifts according to the relation

$$\omega' \cdot n_{p,r}(\omega') = \omega_0 \cdot n_{air}(\omega_0) \tag{1}$$

where red shifts occur when $n_{p,r} > n_{air}$ and blue shifts when $n_{p,r} < n_{air}$. Here, the subscript r denotes the real part. In contrast to the pure space boundary case (Fig. 1B), these dynamically formed time boundaries uniquely enable frequency conversion, a process dictated by the relative time delay between plasma generation and THz wave interaction.

Specifically, let us consider an electromagnetic wave propagating in a homogeneous medium whose permittivity changes abruptly. **E** is the electric field, and **P** is the polarization. By Fourier transforming the evolutions of **E** field and **P** field, it will come to the reduced formulas to describe the propagation property under a lossless case (*29*)

$$c^2k^2\mathbf{E} - \omega'^2\mathbf{E} = \frac{\omega'^2}{\varepsilon_0}\mathbf{P} \tag{2}$$

$$-\omega'^2\mathbf{P} + \omega_s^2\mathbf{P} = \varepsilon_0\omega_p^2\mathbf{E} \tag{3}$$

where $ck = n_{air}\omega_0$ indicates a linear relationship between frequency ω_0 and the wave number k in the air, ω_s is the resonance frequency of the medium, and ω_p is the plasma frequency equaling to $\sqrt{\rho_e e^2/\varepsilon_0 m_e}$ with e and m_e being the charge and mass of an electron and ρ_e being the electron density. Solving ω' will yield the two shifted frequencies $\omega'_{1,2}$ from one single original frequency ω_0, which can be expressed as (*29*, *30*)

$$\omega'_{1,2} = \left[\frac{1}{2}\left(\omega_0^2+\omega_s^2+\omega_p^2\right) \pm \frac{1}{2}\sqrt{\left(\omega_0^2+\omega_s^2+\omega_p^2\right)^2 - 4\omega_s^2\omega_0^2}\right]^{1/2} \tag{4}$$

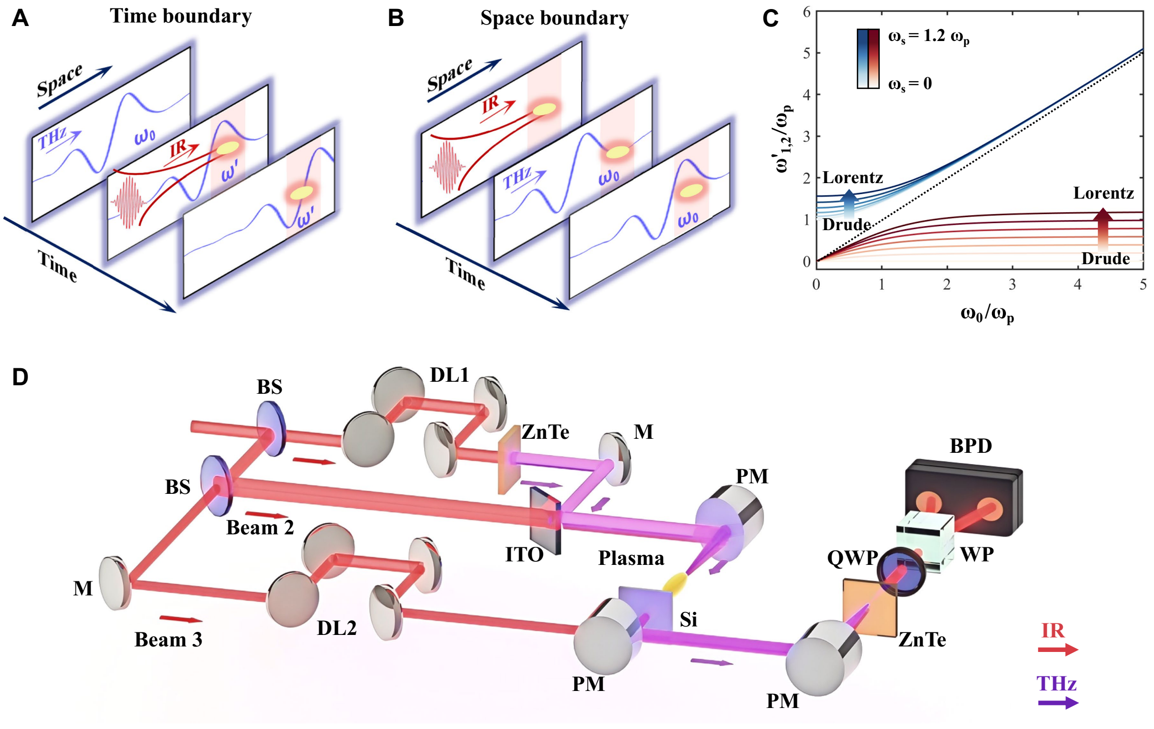

Fig. 1. Overview of the plasma time boundary and the dispersion-dependent frequency conversion. (**A**) THz wave propagates through a plasma time boundary induced by laser ionization. This boundary introduces an abrupt refractive index change in the time domain and shifts the THz frequency from ω_0 to shifted frequencies ω'. (**B**) When plasma is generated before THz arrival, it acts as a pure space boundary with no frequency shift. IR, infrared. (**C**) The frequency shifts from the original frequency ω_0 to shifted frequencies ω' under the lossless case. The blue curves above the dotted lines indicate the blue shifts, whereas the red curves below the dotted lines indicate the red shifts. The curves from light color to the dark color show the trends of frequency shifts from only blue shifts to the bidirectional shifts, with the resonant frequencies ω_s varying from *0* to 1.2 ω_p (spacing with 0.2 ω_p). All the frequencies here are normalized to the plasma frequency ω_p. (**D**) The experimental setup. BS, beam splitter; DL1 (2), delay line 1 (2); ZnTe, zinc telluride crystal; M, mirror; ITO, indium tin oxide glass; PM, parabolic mirror; Si, silicon plate; QWP, quarter wavelength plate; WP, Wollaston prism; BPD, balanced photodiodes.

Note that, when ω_s equals zero or is far less than the other two frequency items (i.e., $\omega_s \ll \omega_0$ or ω_p), this formula will reduce to only one nonzero shifted frequency with $\omega' = \sqrt{\omega_0^2 + \omega_p^2}$.

As shown in Fig. 1C, we compare the frequency shifts under different resonant frequencies ω_s, using blue and red lines to represent the frequency blue shift (upshift) and red shift (downshift), respectively. For the Drude dispersion case with $\omega_s = 0$, the red-shifted frequency conversion (lightest red) faded to zero for all initial frequencies ω_0. Under this condition, only a single frequency shift occurs, namely, frequency blue shift, as observed in most previous experiments and studies. When ω_s is nonzero, bidirectional frequency shifts occur, related to the Lorentzian dispersion caused by the resonance frequency. The figure shows that both blue-shifted and red-shifted frequencies increase with the rising amount of resonance frequency. The corresponding frequency shift can be calibrated as the distance from the shifted frequency to the initial frequency point at the dotted lines. When the resonance frequency is relatively low, the blue-shift magnitude closely resembles the results from Drude dispersion, whereas the red shift yields a shifted frequency very close to zero. Experimentally, such signals may be difficult to observe as these low frequencies could fall outside the permissible frequency measurement range of the equipment. All our discussions here are based on a lossless condition. Actually, numerical analysis of frequency conversion as well as the redistribution of forward and backward amplitudes under lossy Lorentzian dispersion case indicate that damping of the resonance may introduce considerable changes in the imaginary part of the converted frequency, leading to more complex oscillations in the time reflected and time refracted signals when the damping rate is large (*30*).

Experimentally, a Ti:sapphire femtosecond laser pulse (100 fs, 800 nm, 1 kHz, 5.0 mJ) is split into three beams, for THz wave generation, plasma boundary preparation, and the THz wave detection, as shown in Fig. 1D. THz waves generated by beam 1 spatially overlap with the plasma generated by beam 2 using the same parabolic mirror. The transmitted THz waves are electro-optically sampled by beam 3. Switching between the plasma time boundary and space boundary can be achieved by adjusting the time delay between beam 1 and beam 2. The electron density of plasma can be estimated from the traced fluorescence from plasma (*40*). Due to the notable wavelength difference between femtosecond laser pulses and THz waves, the actual interaction volume of plasma is much smaller than the THz wave's focal spot area. This results in relatively weaker interaction between the input THz waves and plasma, and preparing a larger-scale plasma can improve the time-boundary effect and increase the frequency conversion efficiency.

Model for bidirectional frequency conversions

To evaluate the Lorentzian dispersion-induced bidirectional frequency shifting at the plasma time boundary, we propose a simple model that integrates the tunneling ionization and time refraction. When exposed to an intense femtosecond laser, gaseous molecules undergo tunneling ionization. As illustrated in Fig. 2A, by using the molecular Ammosov-Delone-Krainov (MO-ADK) model with the ground-state depletion (*41*), we calculated the tunneling ionization rates and cumulative probabilities of N_2 molecules under a Gaussian pulse (100-fs full width at half maximum) with a peak intensity $\sim 1.7 \times 10^{-4}$ W/cm^2. Tunneling ionization predominantly occurs in bursts synchronized with the laser field's sub-cycle peaks, producing a step-like electron density $\rho_e(t)$ within 100 fs, shorter than the picosecond-scaled THz oscillating period. This step-like increase of the electron density has recently been verified by in situ characterizing the strong field ionization (*42*). It enables an ultrafast time boundary for the propagating THz waves. Note that the time delay t is defined within the pulse envelope, with $t = 0$ corresponding to the envelope peak.

The step-like increase of electron density induces an abrupt, time-varying change in the refractive index experienced by the transmitted THz waves. Assuming that the laser-ionized plasma forms a cylinder with uniform electron density $\rho_e(t)$, the external THz field generates a transient localized field within plasma (*43*). This localized field resonates with the incident THz waves, thereby modifying the refractive index in the THz frequency band. Given the picosecond timescale of the time-boundary interaction, we attribute the electron density solely to tunneling ionization and neglect any further evolution from collisional ionization or electron-particle recombination during the THz-plasma interaction. Under these assumptions, the laser-induced plasma behaves as a dispersive medium with a time-varying refractive index. The transient complex plasma dielectric response during laser excitation can therefore be expressed as (*33*)

$$\varepsilon_p(\omega', t) = 1 - \frac{\omega_p(t)^2}{\omega'^2 - \omega_p(t)^2/2 + i\gamma\omega} \tag{5}$$

where $\omega_p(t) = \sqrt{\rho_e(t)e^2/\varepsilon_0 m_e}$ is the time-dependent plasma frequency and γ is the collisional damping rate (*43*, *33*). The term $\omega_p^2/2$ arises from the screening effect of the electron motions in plasma, as detailed in section S1. Therefore, the Lorentzian resonance frequency $\omega_s = \omega_p/\sqrt{2}$ emerges from the cylindrical boundary conditions of plasma filament and has been experimentally verified across multiple gas types and electron densities (*33*, *43*–*45*). Simulations of plasma with the electron collision included also obtained the frequency downshifts of microwave (*46*), suggesting a similar behavior of electrons. The formula of Eq. 5 allows the calculation of time-dependent plasma refractive index $n_{p,r}(\omega, t) = \mathfrak{Re}\left[\sqrt{\varepsilon_p(\omega, t)}\right]$. As shown in Fig. 2B, the refractive index equals unity across all frequencies for $t < -25$ fs. At $t \simeq -25$ fs, the real part of the refractive index becomes dispersive and deviates from unity, consistent with the ionization sketched in Fig. 2A, where ionization onset occurs at ~ -25 fs. After $t = 25$ fs, the ionization probability reaches its peak, and the electron density, along with the refractive index, becomes time-independent. Figure 2C shows the real and the imaginary parts of the refractive index when the time delay $t = 100$ fs, based on Eq. 5 or a lossless case, respectively. It exhibits a strong resonant centering at $\omega_s \simeq 0.7$ THz, inducing the discrepancy of the calculated refractive indexes around the resonant frequency between the lossy and lossless cases. The tunneling ionization process generates free electrons with zero average initial velocity on attosecond timescales (*47*). Under these passive boundary conditions, the conduction current is conserved across the time boundary, and the electromagnetic energy is partially converted into electron kinetic energy (*27*, *48*).

Within the laser pulse duration, the propagating THz waves undergo frequency shifts from ω_0 to ω' due to the change of refractive index from air to plasma. As shown in Fig. 2D, as the electron density increases, the refractive index variations can cause the original frequency to convert into multiple frequencies following the momentum

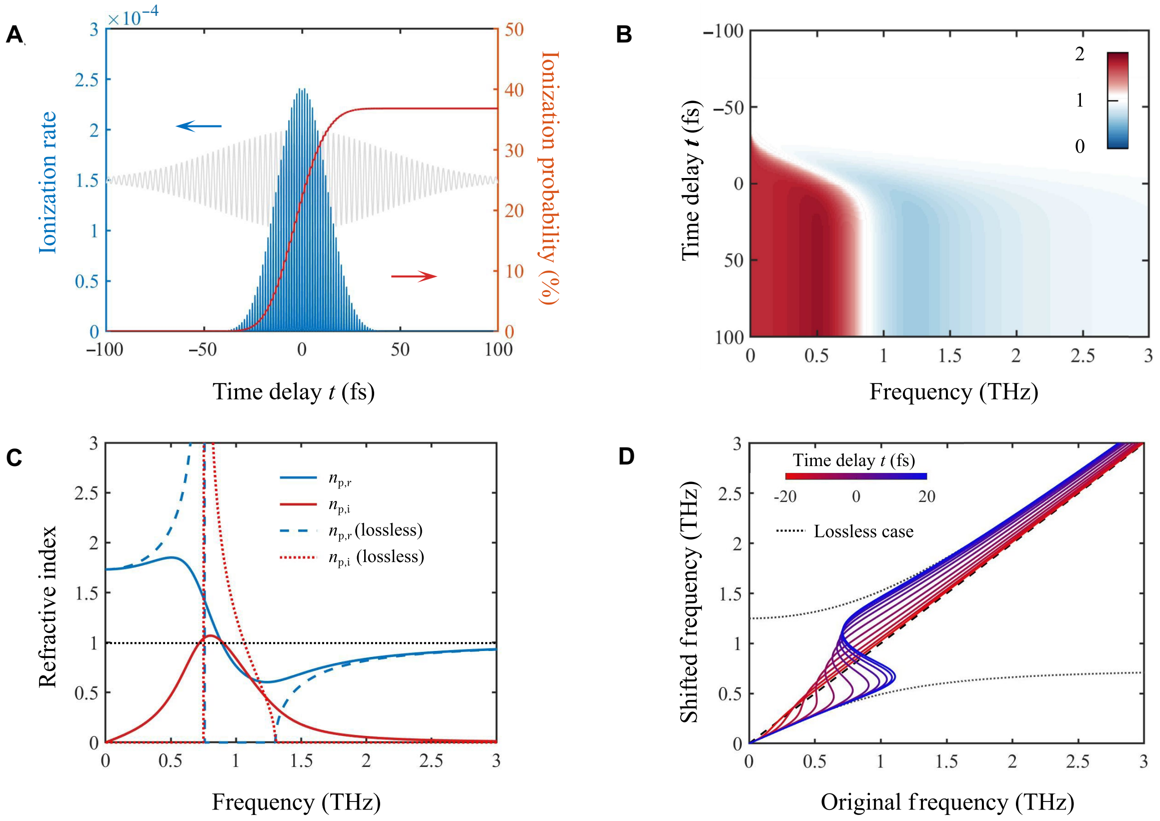

Fig. 2. Theoretical calculations based on the simple-man model. (**A**) Theoretically calculated tunneling ionization rate (left *y* axis, blue solid lines) and the ionization probability (right *y* axis, red solid lines) using the molecular Ammosov-Delone-Krainov (MO-ADK) model (*41*). The gray lines indicate the shape of the incident laser fields. (**B**) The time- and frequency-dependent real part of the refractive index. (**C**) The real parts (blue solid lines) and the imaginary parts (red dashed lines) of the refractive index are presented when the time delay t = 100 fs. The real (blue dashed lines) and the imaginary parts (dotted red lines) for the lossless case are compared. (**D**) Time delay t–dependent frequency variation from the original frequency to the shifted frequency. The zero time delay is set at the peak of the envelope of the laser pulse. The dashed black line indicates cases where the original frequency does not shift. The region below the dashed line corresponds to red shift, whereas the region above corresponds to blue shift. The dotted gray line indicates the lossless condition.

conversion of Eq. 1, producing a bistable frequency variation curve. Here, we select one time moment, the blue curve at $t = 20$ fs, as an example to analyze the frequency conversion behavior. It can be seen that the curve exhibits an "S"-shaped bistable structure with two turning points. As a comparison, we calculate the converted frequencies under the lossless condition by using Eq. 4. The converted frequencies for the lossless case show a split of the up-branch and down-branch, with a frequency gap between 0.75 THz and 1.25 THz corresponding to the zero value in the real parts of the refractive index.

Then, turning to the lossy one, the converted frequency behavior can be categorized into two regions: At the two spectral ends, a single original frequency converts into a single shifted frequency, observed in the low-frequency (0 to 0.7 THz) and high-frequency (>1.1 THz) bands; in the mid-frequency band (0.7 to 1.1 THz), a single original frequency can convert into three shifted frequencies. For the high-frequency and low-frequency bands, amplitude conversion can be determined by calculating the time-refraction coefficients (*3*). For the mid-frequency band, the discrepancy between lossy and lossless cases stems from the correction of the damping term to the real parts of the refractive index. The high-frequency band of the lossy case will progressively overlap with the up-branch of the lossless one, whereas the low-frequency band will progressively overlap with the down-branch. Typically, in bistable structures, the nonstable state between two turning points does not occur due to its instability, exhibiting a nonreciprocal behavior. Here, we assume that the mid-frequency band will not undergo frequency conversion due to the resonant absorption. Therefore, we can use the lossless formula (*30*), considering only the highest and lowest frequencies of the bistable mid-frequency band to obtain the amplitude of time refraction.

THz amplitude variation at the time and space boundary

To visualize the difference between the time and space boundaries, we measured the transmission spectra of THz waves through laser-induced plasma at varying time delay t. As shown in Fig. 3A, the black dotted line represents the incident THz waveform as a reference. The red solid line represents the transmitted THz wave after complete time refraction, where the femtosecond laser and THz wave peaks coincide. The blue dashed line shows the results under the space boundary conditions after plasma formation, with the THz wave delayed about 2 ps after the filamentation. It can be seen that, compared to the incident THz wave, the THz wave interacting with the time boundary undergoes a pulse compression, indicated by the red arrows in the figure, while the THz wave under the space boundary exhibits intensity attenuation. Figure 3B shows the Fourier transformed

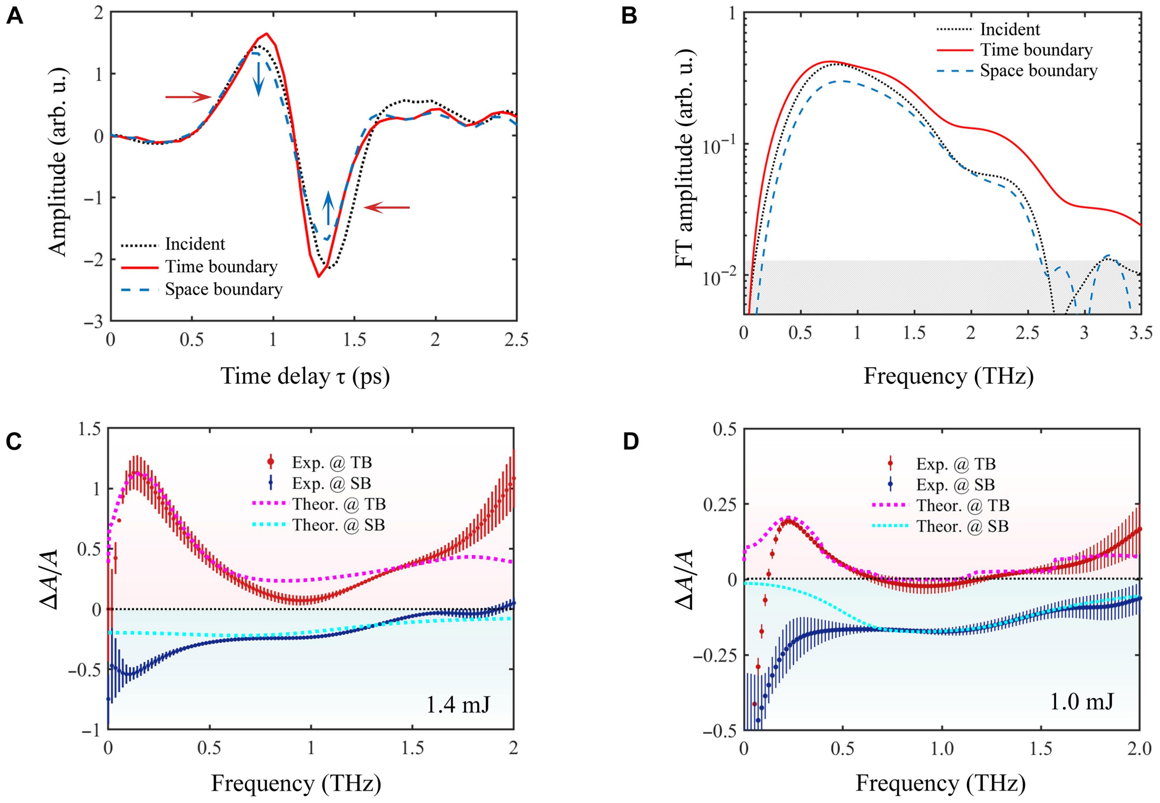

Fig. 3. THz amplitude variation at the time and space boundary. (**A**) Waveforms of the incident THz wave (dotted black lines), transmitted THz wave with a time boundary (red solid lines), and the transmitted THz wave with a space boundary (blue dashed lines). The transmittance of THz waves for the time boundary and the space boundary are 96.8 and 64.4%, respectively. (**B**) The Fourier-transformed (FT) amplitudes of the three THz waveforms in (A). The gray area indicates the noise level. Shifted frequency components appear in the high-frequency region (>1.8 THz) for the time-boundary case compared to the space boundary and the incident THz wave. arb. u., arbitrary units. (**C**) The relative THz amplitude variation ($\Delta A / A$) versus the THz frequencies for the time boundary (red points) and space boundary (blue points) with error bars, as well as the theoretical calculation results of the time boundary (TB; pink dotted lines) and the space boundary (SB; light-blue dotted lines). [(A) to (C)] Carried out under 1.4-mJ input laser energy for plasma preparation. (**D**) The same to (C) under 1.0-mJ laser energy.

THz spectrum of the waveforms in Fig. 3A. The gray shaded area roughly indicates the noise level with a measured signal-to-noise ratio of ~30 dB. The measurements were carried out under a dry environment with humidity of <2%, leading to a relatively smooth THz spectrum. The spectrum of the THz wave interacting with the time boundary exhibits a pronounced spectral extension compared to the incident THz wave, and this frequency-selective broadening effect shows a notable bidirectional trend. In contrast, under the pure space boundary, the THz wave undergoes a broadband resonant absorption, which aligns with the previous works (*33*, *45*, *49*, *50*). It provides definitive proof of shifted frequency generation at the time boundary. The time-boundary spectrum shows distinct amplitude components above 1.8 THz, whereas the space boundary and incident pulse cut off abruptly at 1.8 THz. Actually, a time interface fundamentally implies the generation of shifted frequencies, as established in the theoretical frameworks with $\mu_r = 1$ (*3*). Without a time interface, the system would remain linear and passive, prohibiting this marked high-frequency enhancement.

Figure 3 (C and D) presents the experimental and theoretical results for the relative amplitude variation of the THz wave, when plasma is generated with incident laser energies of 1.4 and 1.0 mJ, respectively. To quantify the time and space boundary effects, here, we define a parameter for the relative THz amplitude modulation, $\Delta A/A = \left(A_{post} - A_{pre}\right)/A_{pre}$, where $A_{pre}(\omega)$ and $A_{post}(\omega)$ denote the THz amplitudes before and after interacting with the plasma at frequency ω. The experimental results in these two figures were obtained by dividing the THz amplitude variations of the time or space boundaries by the incident THz amplitude. The error analysis therein was performed by calculating the mean values and their SDs of each $\Delta A/A$ from three independent measurements. The theoretical results were obtained using the incident THz amplitude and the calculated amplitude variation accordingly. Details on how to calculate the amplitude variation of the time boundary are provided in section S2, whereas those for the space boundary are provided in section S3.

As shown in Fig. 3 (C and D), the color-coded regions directly show this frequency-selective enhancement and attenuation. Positive values of $\Delta A/A$ indicate amplitude enhancement, whereas negative values indicate attenuation. For the time boundary under 1.4-mJ case, THz amplitude enhancements occur at both ends of the measured THz spectra, with maximum gains of ~110% at 0.14 THz in the low-frequency wing and ~100% at 2.0 THz in the high-frequency wing, which exceeds the gain efficiency of about 12% from photon-excited silicon metasurface (*51*). This dual-band response suggests the bidirectional frequency shifting, i.e., concurrent blue- and red-shifted THz spectrum components, in contrast with the unidirectional shifts

reported previously. The lowest gain of ~7% occurs at around 1.0 THz in the middle frequency range, indicating a unique time-boundary effect in the air-to-plasma phase transition. This gain property here implies a nonconventional frequency generation around the resonant frequencies in the time varying Lorentzian dispersion system (*31*). The space boundary interaction merely causes attenuation across the entire 0.3- to 2.0-THz spectral range, distinguishing the two regimes between the time and space boundaries. In the calculation, only the interface reflections and the plasma absorption are considered. The general trends of calculations show good consistency with experiments, while the discrepancies between theory and experiment in the low and high frequency ends likely stem from the limited THz generation and electro-optic sampling efficiency at these frequencies.

When the laser intensity for plasma generation is changed to 1.0 mJ in Fig. 3D, the plasma density as well as the plasma-THz interaction cross section will be decreased. This results in a notable reduction in the amplitude of the frequency shift, such as the relative amplitude variation on the low-frequency and high-frequency wings decreasing from over 100% to less than 25%, although the overall trend remains unchanged. As can be seen in Fig. 3 (C and D), when the pump energy increases from 1.0 to 1.4 mJ, the peak of the low-frequency amplitude variation progressively shifts from 0.21 to 0.13 THz ($\Delta f \approx -0.08$ THz). This red-shift trend demonstrates that a higher plasma density leads to a larger change in the refractive index, confirming the intensity-dependent nature of plasma time-boundary effects instead of the photon acceleration effect (see section S4 for details).

Time delay–dependent frequency conversion at the time boundary

Based on the proposed simple model, the time delay t–dependent amplitude variations $\Delta A/A$ induced by the time boundaries can be calculated during the rising up process of electron density, as sketched in Fig. 4A. The minimal walk-off ensures the temporal overlap between the infrared pump and THz wave throughout the interaction region, as detailed in section S5.

It can be observed from Fig. 4B that, when the plasma generation precedes the arrival of the THz wave, the relative amplitude variation of the THz wave exhibits a pronounced absorption, demonstrating the space boundary behavior. When the plasma is generated during or after the arrival of the THz wave, the refractive index experienced undergoes an abrupt change, leading to the bidirectional frequency shifts. Notably, as the contrast in refractive index increases, the bidirectional frequency shift becomes progressively more pronounced. When the time delay $t = 100$ fs, the transmitted THz wave is able to "witness" the entire process of air-to-plasma phase transition corresponding to the largest contrast of the refractive index. Theoretical results in Fig. 4, based on our simple model, can qualitatively reproduce the experimental observations, including the bidirectional frequency shifts and the transition from space to time-boundary effects.

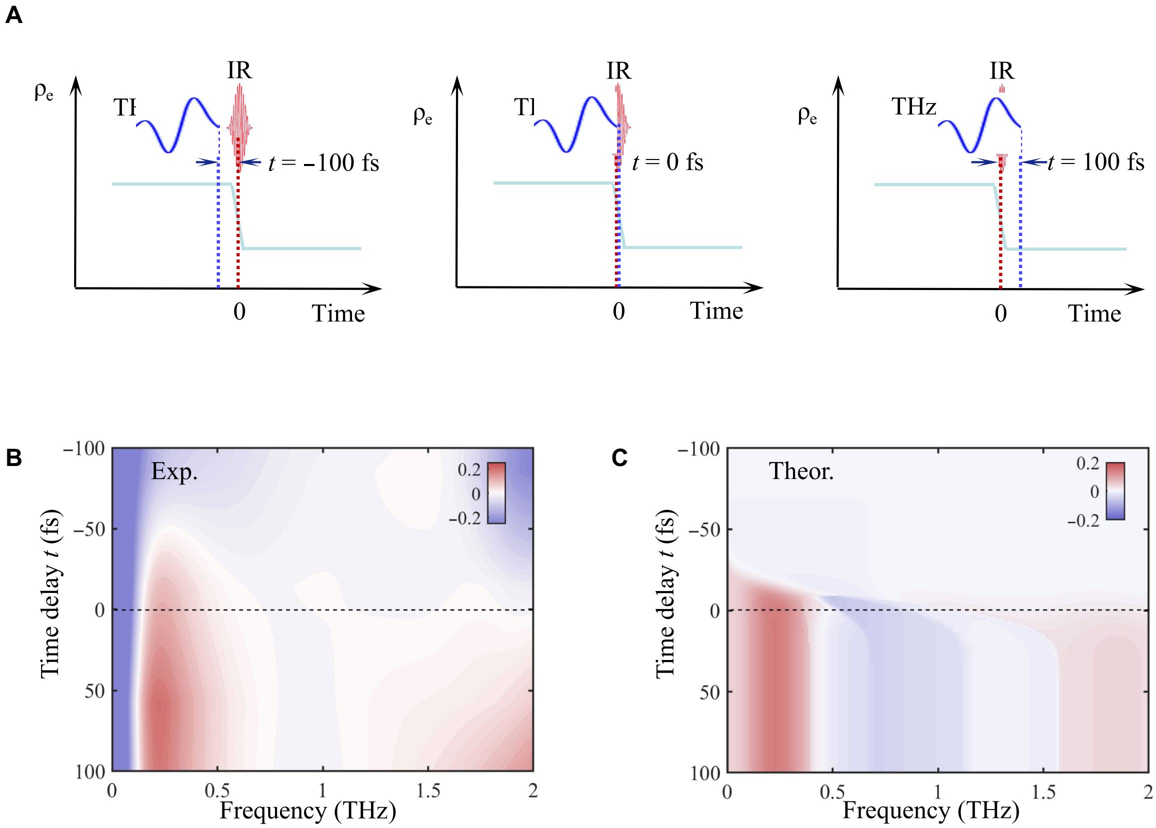

Fig. 4. Time-dependent variations of the relative THz amplitude. (**A**) The temporal coordinates in calculating the time-boundary–induced THz amplitude variation. *t* indicates the time delay between the THz waves and the femtosecond laser pulse (plasma generation). From left to right, we show how the THz wave interacts with the plasma time boundary at varying time delay *t*. For the calculation, we only consider the time delay *t* ranging from −100 fs to 100 fs. IR, infrared. (**B**) The experimental time delay *t*–dependent relative THz amplitude variations (ΔA / A) under 1.0-mJ laser inputs. (**C**) The theoretical calculation results of (B).

For the THz component above the resonance frequency, it propagates through the plasma with low loss, behaving like ordinary transmitted waves in a dielectric medium. Note that low frequencies of THz waves (below the resonance) are confined within the ring-shaped region of the plasma cylinder, propagating and coupling out effectively like guided plasma modes (details in section S6). The extremely low-frequency waves (<0.15 THz) concentrate on the plasma ring's outer edge, where energy leakage reduces the transmission efficiency, and large divergence angles make the collection by off-axis parabolic mirrors inefficient. These two factors explain the suppression of extremely low-frequency components observed in Fig. 4B. For frequencies exceeding 0.1 THz, where the THz wavelength is substantially smaller than the length of plasma channel, these confinement and edge effects become negligible.

DISCUSSIONS

Time-varying media with tunable refractive index modulation present emerging frontiers in photonic control. In this work, we establish laser-induced air-to-plasma phase transitions as a promising approach for dynamic refractive index engineering. These ultrafast phase transitions enable precise spectral control of electromagnetic waves, offering a novel paradigm for time-varying photonics. Structural resonance–induced nearly unity refractive index contrast with Lorentzian-like dispersion facilitates efficient frequency shifting, which is challenging to achieve in nondispersive or Drude conditions.

Because the plasma model used in this work is relatively simple, it will benefit from establishing a more detailed and accurate refractive index model in understanding and optimizing the time-varying dynamics. Further efforts in experiment could explore the use of few-cycle or attosecond pulses (*52*, *53*) to create faster time boundaries for near-infrared or visible light, generate transient localized dipoles in plasma (*54*) to amplify shifted frequencies, and expand the plasma volume to reveal time reflection effects. Time reflection signals in this work remained below the detection threshold, limited by weak conversion efficiency and propagation loss. Another potential extension of the current work is to realize a photonic time crystal in plasma platforms due to the ~10-ns typical recombination time (*55*). Gas engineering (e.g., O_2/SF_6 mixtures) can reduce this to <200 ps (*56*), thus enabling high-frequency periodic modulation.

The present temporal modulation approach provides additional opportunities for air-based electromagnetic wave modulation (*57*). As summarized in Table 1, plasma time boundaries uniquely combine strong frequency conversion with negligible propagation loss in the THz range (more details in section S7), offering a clear advantage over indium tin oxide (ITO)–based platforms that excel in the near-infrared (~240 to 300 THz) but suffer high reflection losses at THz frequencies. The significance of this work extends beyond demonstrating bidirectional frequency conversion: It establishes plasma as a reconfigurable photonic platform capable of femtosecond-scale temporal material modulation. Unlike solid-state materials such as ITO, the plasma's geometry, density profile, and spatial extent can be flexibly tailored within optical resolution limits, enabling dynamic control over transient photonic structures unattainable in fixed-geometry films. This reconfigurability allows air—a ubiquitous, readily available medium—to be tuned across a broad frequency range by adjusting the plasma frequency via ionization. Future plasma-based platforms thus promise to enable richer physical phenomena and more versatile applications. By modulating both spatial and temporal properties of light, complex transient photonic structures can be dynamically sculpted with exceptional flexibility (*58*, *59*).

In conclusion, we experimentally and theoretically demonstrate the bidirectional frequency conversions of THz waves from an ultrafast laser-induced air-to-plasma phase transition. This phenomenon corresponds to the Lorentzian dispersion of the created plasma with a structural resonance, which is different from the typical Drude or dispersionless platform. Using the MO-ADK model and incorporating refractive index variations, our theoretical calculations accurately reproduce the spectral changes induced by time boundaries at most frequencies. These changes contrast with the monotonous broad-spectrum attenuation observed under space boundaries. Our findings deepen the understanding of light-matter interactions at extreme time gradients and lay the groundwork for photonic time crystals and spatiotemporal mixing modulation. Moreover, this phase-transition–based approach, analogous to hollow-fiber self-compression (*60*) but distinguished by its precise temporal and spatial control, may enable more efficient frequency conversion, pulse compression, soliton formation, and high-energy electron generation in future ultrafast photonic systems (*61*–*64*).

MATERIALS AND METHODS

Experimental details

The experiment was implemented with a commercial Ti:sapphire laser system (100 fs, 800 nm, 5.4 mJ, 1 kHz). The output femtosecond laser pulse was split into three beams for THz wave generation, plasma formation, and electro-optic sampling, respectively. Beam 1 passed through a 1-mm-thick (110)–cut zinc telluride (ZnTe) crystal to produce THz waves, which then sequentially reflected by a mirror and a 0.5-mm-thick ITO glass. This ITO glass serves as a beam combiner, reflecting the THz wave while transmitting the 800-nm pulse. The pump intensity incident on the ITO is less than 10 GW/

Table 1. Comparison of time-varying platforms. ENZ, epsilon-near-zero; Freq., frequency; Ref., reference; NA, not applicable.

Platform	Material	Mechanism	n_0	Max Δn	Freq. (THz)	$\Delta f/f$	Ref.
Air-plasma	Plasma	Time refraction	1	0.8/0.4	0.5/1.1	44%/67%	This work
ENZ	ITO	Time refraction	~0.42	NA	242	~9%	(*28*)
ENZ	TCO	Time refraction	0.53	~0.5	250	~3%	(*24*)
Metasurface	Silicon	Time refraction	3.42	~0.06	0.58	1.7%	(*51*)
Metasurface	GaAs	Time refraction	3.6	~0.14	293.9	4%	(*66*)

cm^2, which does not generate measurable nonlinear effects. Beam 2 copropagated with the reflected THz waves and ionized the gaseous molecules at the focal point of an off-axis parabolic mirror with a 7.62-cm focal length. A high-resistance silicon wafer was placed after the focal point, blocking the femtosecond laser while transmitting the THz wave. Beam 3 was focused onto a 1-mm-thick ZnTe crystal along with the interacting THz wave for electro-optic sampling of the transmitted THz waveform. The ZnTe detector has a limited bandwidth within 3 THz, preventing the measurement of high-frequency components. Alternative detection schemes, such as the air-biased coherent detection method (*65*), can offer broader bandwidth to obtain high-frequency end. By adjusting the time delay t between beam 1 and beam 2, we could switch between forming time and space boundaries. The temporal resolution of time delay t is 10 fs in the experiment due to the micrometer-scaled accuracy of the translation stage (Newport, XMS100-S).

Supplementary Materials

This PDF file includes:
Sections S1 to S7
Figs. S1 to S4
References

REFERENCES

1. J. T. Mendonça, *Theory of Photon Acceleration* (Institute of Physics Publishing, 2001).
2. B. W. Plansinis, W. R. Donaldson, G. P. Agrawal, What is the temporal analog of reflection and refraction of optical beams? *Phys. Rev. Lett.* **115**, 183901 (2015).
3. E. Galiffi, R. Tirole, S. Yin, H. Li, S. Vezzoli, P. A. Huidobro, M. G. Silveirinha, R. Sapienza, A. Alù, J. B. Pendry, Photonics of time-varying media. *Adv. Photonics* **4**, 014002 (2022).
4. L. Bar-Hillel, A. Dikopoltsev, A. Kam, Y. Sharabi, O. Segal, E. Lustig, M. Segev, Time refraction and time reflection above critical angle for total internal reflection. *Phys. Rev. Lett.* **132**, 263802 (2024).
5. M. M. Asgari, P. Garg, X. Wang, M. S. Mirmoosa, C. Rockstuhl, V. Asadchy, Theory and applications of photonic time crystals: A tutorial. *Adv. Opt. Photonics* **16**, 958–1063 (2024).
6. Z. Hayran, F. Monticone, A resonant tone for photonic time crystals. *Nat. Photonics* **19**, 126–128 (2025).
7. M. Lyubarov, Y. Lumer, A. Dikopoltsev, E. Lustig, Y. Sharabi, M. Segev, Amplified emission and lasing in photonic time crystals. *Science* **377**, 425–428 (2022).
8. X. Wang, P. Garg, M. S. Mirmoosa, A. G. Lamprianidis, C. Rockstuhl, V. S. Asadchy, Expanding momentum bandgaps in photonic time crystals through resonances. *Nat. Photonics* **19**, 149–155 (2025).
9. N. Konforty, M.-I. Cohen, O. Segal, Y. Plotnik, V. M. Shalaev, M. Segev, Second harmonic generation and nonlinear frequency conversion in photonic time-crystals. *Light Sci. Appl.* **14**, 152 (2025).
10. E. Galiffi, Y.-T. Wang, Z. Lim, J. B. Pendry, A. Alù, P. A. Huidobro, Wood anomalies and surface-wave excitation with a time grating. *Phys. Rev. Lett.* **125**, 127403 (2020).
11. Y. Sharabi, E. Lustig, M. Segev, Disordered photonic time crystals. *Phys. Rev. Lett.* **126**, 163902 (2021).
12. J. Kim, D. Lee, S. Yu, N. Park, Unidirectional scattering with spatial homogeneity using correlated photonic time disorder. *Nat. Phys.* **19**, 726–732 (2023).
13. Z. Dong, X. Chen, L. Yuan, Extremely narrow band in moiré photonic time crystal. *Phys. Rev. Lett.* **135**, 033803 (2025).
14. Y. Sharabi, A. Dikopoltsev, E. Lustig, Y. Lumer, M. Segev, Spatiotemporal photonic crystals. *Optica* **9**, 585–592 (2022).
15. X. Wang, M. S. Mirmoosa, V. S. Asadchy, C. Rockstuhl, S. Fan, S. A. Tretyakov, Metasurface-based realization of photonic time crystals. *Sci. Adv.* **9**, eadg7541 (2023).
16. W. Jaffray, S. Stengel, F. Biancalana, C. Fruhling, M. Ozlu, M. Scalora, A. Boltasseva, V. Shalaev, M. Ferrera, Spatio-spectral optical fission in time-varying subwavelength layers. *Nat. Photonics* **19**, 558–566 (2025).
17. H. Moussa, G. Xu, S. Yin, E. Galiffi, Y. Ra'di, A. Alú, Observation of temporal reflection and broadband frequency translation at photonic time interfaces. *Nat. Phys.* **19**, 863–868 (2023).
18. T. Jones, A. Kildishev, M. Segev, D. Peroulis, Time-reflection of microwaves by a fast optically-controlled time-boundary. *Nat. Commun.* **15**, 6786 (2024).
19. A. Nishida, N. Yugami, T. Higashiguchi, T. Otsuka, F. Suzuki, M. Nakata, Y. Sentoku, R. Kodama, Experimental observation of frequency up-conversion by flash ionization. *Appl. Phys. Lett.* **101**, 161118 (2012).
20. F. Miyamaru, C. Mizuo, T. Nakanishi, Y. Nakata, K. Hasebe, S. Nagase, Y. Matsubara, Y. Goto, J. Pérez-Urquizo, J. Madéo, K. M. Dani, Ultrafast frequency-shift dynamics at temporal boundary induced by structural-dispersion switching of waveguides. *Phys. Rev. Lett.* **127**, 053902 (2021).
21. M. Z. Alam, I. D. Leon, R. W. Boyd, Large optical nonlinearity of indium tin oxide in its epsilon-near-zero region. *Science* **352**, 795–797 (2016).
22. R. Tirole, S. Vezzoli, E. Galiffi, I. Robertson, D. Maurice, B. Tilmann, S. A. Maier, J. B. Pendry, R. Sapienza, Double-slit time diffraction at optical frequencies. *Nat. Phys.* **19**, 999–1002 (2023).
23. J. Bohn, T. S. Luk, S. Horsley, E. Hendry, Spatiotemporal refraction of light in an epsilon-near-zero indium tin oxide layer: Frequency shifting effects arising from interfaces. *Optica* **8**, 1532–1537 (2021).
24. E. Lustig, O. Segal, S. Saha, E. Bordo, S. N. Chowdhury, Y. Sharabi, A. Fleischer, A. Boltasseva, O. Cohen, V. M. Shalaev, M. Segev, Time-refraction optics with single cycle modulation. *Nanophotonics* **12**, 2221–2230 (2023).
25. Y. Lu, X. Zhang, H. Qiu, L. Niu, X. Chen, Q. Xu, W. Zhang, S. Zhang, J. Han, Ultrafast temporal modulation of terahertz generation at an optically pumped ITO interface. *Optica* **12**, 1035–1043 (2025).
26. F. Morgenthaler, Velocity modulation of electromagnetic waves. *IRE Trans. Microw. Theory Tech.* **6**, 167–172 (1958).
27. E. Galiffi, D. M. Solís, S. Yin, N. Engheta, A. Alù, Electrodynamics of photonic temporal interfaces. *Light Sci. Appl.* **14**, 338 (2025).
28. Y. Zhou, M. Z. Alam, M. Karimi, J. Upham, O. Reshef, C. Liu, A. E. Willner, R. W. Boyd, Broadband frequency translation through time refraction in an epsilon-near-zero material. *Nat. Commun.* **11**, 2180 (2020).
29. K. Qu, Q. Jia, M. R. Edwards, N. J. Fisch, Theory of electromagnetic wave frequency upconversion in dynamic media. *Phys. Rev. E* **98**, 023202 (2018).
30. D. M. Solís, R. Kastner, N. Engheta, Time-varying materials in the presence of dispersion: Plane-wave propagation in a Lorentzian medium with temporal discontinuity. *Photonics Res.* **9**, 1842–1853 (2021).
31. C. Rizza, M. A. Vincenti, G. Castaldi, A. Contestabile, V. Galdi, M. Scalora, Harnessing the natural resonances of time-varying dispersive interfaces. *Phys. Rev. Lett.* **133**, 186902 (2024).
32. P. Garg, E. Almpanis, L. Zimmer, J. D. Fischbach, X. Wang, M. S. Mirmoosa, M. Nyman, N. Stefanou, N. Papanikolaou, V. Asadchy, C. Rockstuhl, Photonic time crystals assisted by quasi-bound states in the continuum. arXiv:2507.15644 [physics.optics] (2025).
33. Z. Zheng, Y. Huang, Q. Guo, C. Meng, Z. Lu, X. Wang, J. Zhao, C. Meng, D. Zhang, J. Yuan, Z. Zhao, Filament characterization via resonance absorption of terahertz wave. *Phys. Plasmas* **24**, 103303 (2017).
34. S. P. Kuo, Frequency up-conversion of microwave pulse in a rapidly growing plasma. *Phys. Rev. Lett.* **65**, 1000–1003 (1990).
35. J. M. Dias, C. Stenz, N. Lopes, X. Badiche, F. Blasco, A. Dos Santos, L. Oliveira e Silva, A. Mysyrowicz, A. Antonetti, J. T. Mendonça, Experimental evidence of photon acceleration of ultrashort laser pulses in relativistic ionization fronts. *Phys. Rev. Lett.* **78**, 4773–4776 (1997).
36. I. Geltner, Y. Avitzour, S. Suckewer, Picosecond pulse frequency upshifting by rapid free-carrier creation in ZnSe. *Appl. Phys. Lett.* **81**, 226–228 (2002).
37. M. R. Edwards, K. Qu, Q. Jia, J. M. Mikhailova, N. J. Fisch, Cascaded chirped photon acceleration for efficient frequency conversion. *Phys. Plasmas* **25**, 053102 (2018).
38. A. J. Howard, D. Turnbull, A. S. Davies, P. Franke, D. H. Froula, J. P. Palastro, Photon acceleration in a flying focus. *Phys. Rev. Lett.* **123**, 124801 (2019).
39. R. T. Sandberg, A. G. R. Thomas, Photon acceleration from optical to XUV. *Phys. Rev. Lett.* **130**, 085001 (2023).
40. X. Xu, Y. Huang, Z. Zhang, J. Liu, J. Lou, M. Gao, S. Wu, G. Fang, Z. Zhao, Y. Chen, Z. Sheng, C. Chang, Laser-chirp controlled terahertz wave generation from air plasma. *Chin. Phys. Lett.* **40**, 045201 (2023).
41. X. M. Tong, Z. X. Zhao, C. D. Lin, Theory of molecular tunneling ionization. *Phys. Rev. A* **66**, 033402 (2002).
42. N. Shlomo, E. Frumker, In situ characterization of laser-induced strong field ionization phenomena. *Light Sci. Appl.* **14**, 166 (2025).
43. Z. Mics, F. Kadlec, P. Kužel, P. Jungwirth, S. E. Bradforth, V. A. Apkarian, Nonresonant ionization of oxygen molecules by femtosecond pulses: Plasma dynamics studied by time-resolved terahertz spectroscopy. *J. Chem. Phys.* **123**, 104310 (2005).
44. Z. Mics, P. Kužel, P. Jungwirth, S. E. Bradforth, Photoionization of atmospheric gases studied by time-resolved terahertz spectroscopy. *Chem. Phys. Lett.* **465**, 20–24 (2008).
45. Y. Huang, Z. Xiang, X. Xu, J. Zhao, J. Liu, R. Wang, Z. Zhang, Z. Lü, D. Zhang, C. Chang, J. Yuan, Z. Zhao, Localized-plasma-assisted rotational transitions in the terahertz region. *Phys. Rev. A* **103**, 033109 (2021).
46. S. P. Kuo, A. Ren, G. Schmidt, Frequency downshift in rapidly ionizing media. *Phys. Rev. E* **49**, 3310–3315 (1994).
47. S. Undurti, H. Xu, X. Wang, A. Noor, W. Wallace, N. Douguet, A. Bray, I. Ivanov, K. Bartschat, A. Kheifets, R. Sang, I. Litvinyuk, Attosecond angular streaking and tunnelling time in atomic hydrogen. *Nature* **568**, 75–77 (2019).

48. H. Mehrpour Bernety, D. K. Kalluri, M. A. Cappelli, Proper treatment of energy and momentum in time-modulated plasmas. *Phys. Rev. E* **113**, 025204 (2026).
49. X.-K. Wang, J.-S. Ye, W.-F. Sun, P. Han, L. Hou, Y. Zhang, Terahertz near-field microscopy based on an air-plasma dynamic aperture. *Light Sci. Appl.* **11**, 129 (2022).
50. J. Zhao, F. Zhu, Y. Han, Q. Wang, L. Lao, X. Li, Y. Peng, Y. Zhu, Light-guiding-light-based temporal integration of broadband terahertz pulses in air. *APL Photonics* **8**, 106107 (2023).
51. G. Xu, H. Xing, D. Lu, J. Fan, Z. Xue, P. P. Shum, L. Cong, Linear terahertz frequency conversion in a temporal-boundary metasurface. *Laser Photonics Rev.* **18**, 2301294 (2024).
52. I. Ahmad, L. Bergé, Z. Major, F. Krausz, S. Karsch, S. A. Trushin, Redshift of few-cycle infrared pulses in the filamentation regime. *New J. Phys.* **13**, 093005 (2011).
53. H. Kim, C. M. Kim, K. H. Pae, K. T. Kim, Isolated attosecond pulses generated from a relativistic plasma mirror via noncollinear gating. *Phys. Rev. Res.* **7**, 013216 (2025).
54. X. Qu, Y. Huang, B. Zhou, M. Gao, J. Lou, Y. Feng, Z. Zhao, C. Chang, A. P. Shkurinov, J. Verboncoeur, Ultrafast plasma-based terahertz modulator. *Optica* **11**, 1478–1481 (2024).
55. J. Papeer, D. Gordon, P. Sprangle, M. Botton, A. Zigler, Temporal evolution of femtosecond laser induced plasma filament in air and N_2. *Appl. Phys. Lett.* **103**, 244102 (2013).
56. T. Kampfrath, D. O. Gericke, L. Perfetti, P. Tegeder, M. Wolf, C. Frischkorn, Long- and short-lived electrons with anomalously high collision rates in laser-ionized gases. *Phys. Rev. E* **76**, 066401 (2007).
57. Y. Schrödel, C. Hartmann, J. Zheng, T. Lang, M. Steudel, M. Rutsch, S. H. Salman, M. Kellert, M. Pergament, T. Hahn-Jose, S. Suppelt, J. H. Dörsam, A. Harth, W. P. Leemans, F. X. Kärtner, I. Hartl, M. Kupnik, C. M. Heyl, Acousto-optic modulation of gigawatt-scale laser pulses in ambient air. *Nat. Photonics* **18**, 54–59 (2024).
58. X.-B. Zhang, S.-M. Weng, H. Ai, X. Qiao, J.-K. Xue, Z.-M. Sheng, Photonic rabi oscillations in defective plasma photonic crystals. *Phys. Rev. Lett.* **135**, 015101 (2025).
59. G. Lehmann, K. H. Spatschek, Transient plasma photonic crystals for high-power lasers. *Phys. Rev. Lett.* **116**, 225002 (2016).
60. N. L. Wagner, E. A. Gibson, T. Popmintchev, I. P. Christov, M. M. Murnane, H. C. Kapteyn, Self-compression of ultrashort pulses through ionization-induced spatiotemporal reshaping. *Phys. Rev. Lett.* **93**, 173902 (2004).
61. Y. Pan, M.-I. Cohen, M. Segev, Superluminal *k*-gap solitons in nonlinear photonic time crystals. *Phys. Rev. Lett.* **130**, 233801 (2023).
62. Y. Jang, B. Oh, E. Kim, J. Rho, Bidirectional asymmetric frequency conversion in nonlinear phononic crystals. *Phys. Rev. Lett.* **135**, 036603 (2025).
63. J. Dong, S. Zhang, H. He, H. Li, J. Xu, Nonuniform wave momentum band gap in biaxial anisotropic photonic time crystals. *Phys. Rev. Lett.* **134**, 063801 (2025).
64. J. Ying, L. Liu, L. Zheng, D. Su, X. He, J. Ma, H. Xuan, D. Zhang, High-energy subcycle electron emission driven by spatiotemporally confined THz fields. *Phys. Rev. X* **15**, 021095 (2025).
65. N. Karpowicz, J. Dai, X. Lu, Y. Chen, M. Yamaguchi, H. Zhao, X.-C. Zhang, L. Zhang, C. Zhang, M. Price-Gallagher, C. Fletcher, O. Mamer, A. Lesimple, K. Johnson, Coherent heterodyne time-domain spectrometry covering the entire "terahertz gap". *Appl. Phys. Lett.* **92**, 011131 (2008).
66. M. R. Shcherbakov, S. Liu, V. Zubyuk, A. Vaskin, P. Vabishchevich, G. Keeler, T. Pertsch, T. Dolgova, I. Staude, I. Brener, A. Fedyanin, Ultrafast all-optical tuning of direct-gap semiconductor metasurfaces. *Nat. Commun.* **8**, 17 (2017).
67. B. M. Smirnov, M. I. Chibisov, The breaking up of atomic particles by an electric field and by electron collisions. *Sov. Phys. JETP* **22**, 585–592 (1966).
68. M.-V. Ammosov, N.-B. Delone, V.-P. Krainov, Tunnel ionization of complex atoms and atomic ions in electromagnetic field. *Sov. Phys. JETP* **64**, 1191–1194 (1986).
69. J. M. Dias, N. C. Lopes, L. O. Silva, G. Figueira, J. T. Mendonça, C. Stenz, F. Blasco, A. Dos Santos, A. Mysyrowicz, Photon acceleration of ultrashort laser pulses by relativistic ionization fronts. *Phys. Rev. E* **66**, 056406 (2002).
70. S. Fu, B. Groussin, Y. Liu, A. Mysyrowicz, V. Tikhonchuk, A. Houard, Steering laser-produced THz radiation in air with superluminal ionization fronts. *Phys. Rev. Lett.* **134**, 045001 (2025).
71. S. C. Wilks, J. M. Dawson, W. B. Mori, Frequency up-conversion of electromagnetic radiation with use of an overdense plasma. *Phys. Rev. Lett.* **61**, 337–340 (1988).
72. R. Loudon, The propagation of electromagnetic energy through an absorbing dielectric. *J. Phys. A: Gen. Phys.* **3**, 233–245 (1970).
73. L. Brillouin, *Wave Propagation and Group Velocity*. (Academic Press, 1960).
74. K. Oughstun, C. Balictsis, Gaussian pulse propagation in a dispersive, absorbing dielectric. *Phys. Rev. Lett.* **77**, 2210–2213 (1996).
75. J. Zhao, Y. Zhang, Z. Wang, W. Chu, B. Zeng, W. Liu, Y. Cheng, Z. Xu, Propagation of terahertz wave inside femtosecond laser filament in air. *Laser Phys. Lett.* **11**, 095302 (2014).
76. J. Zhao, W. Chu, Z. Wang, Y. Peng, C. Gong, L. Lin, Y. Zhu, W. Liu, Y. Cheng, S. Zhuang, Z. Xu, Strong spatial confinement of terahertz wave inside femtosecond laser filament. *ACS Photonics* **3**, 2338–2343 (2016).

Acknowledgments: We thank the helpful discussions with M. Chen and X. Zhang. **Funding:** This work was supported by the National Natural Science Foundation of China (nos. 12225511, 12174449, T2241002, and 62541116); the Fundamental Research Funds for the Central Universities, China (no. 3072024WD2603); the Chinese Academy of Sciences (project nos. E4BA270100, E4Z127010F, E4Z6270100, and E53327020D); and the Finnish Foundation for Technology Promotion. **Author contributions:** Conceptualization: Y.H., C.S., X.W., C.C., and V.A. Methodology: Y.H., M.G., J.L., Z.Z., C.S., X.W., and C.C. Investigation: X.Q., M.G., Y.H., C.S., and Z.Z. Visualization: Y.H. and C.C. Supervision: Y.H., C.S., and C.C. Resources: Y.H. and C.C. Funding acquisition: Y.H., C.S., X.W., and C.C. Data curation: A.X., M.G., and J.L. Validation: Y.H., M.G., C.S., X.W., and C.C. Formal analysis: Y.H., M.G., B.Z., C.S., and V.A. Software: Y.H. and B.Z. Project administration: Y.H., C.S., and C.C. Writing—original draft: Y.H., B.Z., M.G., C.S., X.W., C.C., and V.A. Writing—review and editing: Y.H., B.Z., C.S., C.C., and V.A. **Competing interests:** The authors declare that they have no competing interests. **Data, code, and materials availability:** All data and code needed to evaluate and reproduce the results in the paper are present in the paper and/or the Supplementary Materials. This study did not generate new materials.

Submitted 22 October 2025
Accepted 25 June 2026
Published 31 July 2026
10.1126/sciadv.aed2916
